# Biorefinery and Valorization Strategies for Sugarcane Bagasse: Integrating Food, Health, Economic, and Industrial Applications

**DOI:** 10.1002/fsn3.71262

**Published:** 2025-12-15

**Authors:** Desye Alemu Teferi, Messenbet Geremew Kassa, Mikru Tesfa Belachew, Eshetie Gelagay Erku

**Affiliations:** ^1^ College of Agriculture, Food, Climate Science, Injibara University Injibara Ethiopia

**Keywords:** bioethanol, circular bioeconomy, dietary fiber, functional foods, lignocellulosic biomass, nanocellulose

## Abstract

Sugarcane bagasse, a fibrous by‐product of sugar extraction, is a renewable lignocellulosic biomass with great potential for sustainable value addition across energy, food, health, and industrial sectors. This review summarizes recent advances, key performance metrics, and research gaps in the biorefinery and valorization of sugarcane bagasse. Optimized pretreatment and fermentation have achieved bioethanol yields of 3.7% v/v and methane production of 347.6 mL CH_4_/g volatile solids, highlighting its bioenergy potential. Bagasse‐derived cellulose and nanocellulose enable biodegradable packaging with excellent mechanical and barrier properties, reducing reliance on plastics. In food and feed, bagasse flour increases dietary fiber by 32.39%, improves gut health, and enhances livestock productivity. Additional applications include pollutant adsorption, composite reinforcement, soil enrichment, and enzyme production. However, large‐scale utilization is hindered by high costs and regulatory challenges. Future research should emphasize green technologies, safety assessments, and integration into circular bio‐economy frameworks.

## Graphical abstract

The review presents an integrated biorefinery approach that links sugarcane bagasse utilization across food, health, and industrial sectors. It explores the potential of bagasse‐derived fibers and compounds in functional foods and edible coatings. It highlights the antioxidant, antimicrobial, and prebiotic properties of sugarcane bagasse for disease prevention and gut health. It showcases eco‐friendly methods for efficient and sustainable value recovery. It positions sugarcane bagasse as a cornerstone for zero‐waste, circular bioeconomies.

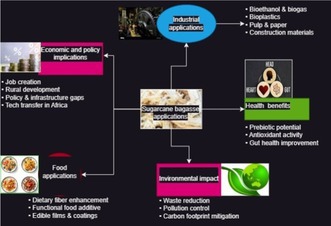

## 1 Introduction

Sugarcane is one of the world's most important agro‐industrial crops, widely cultivated across tropical and subtropical regions. With an estimated annual production of 1.6 billion tons, sugarcane processing generates approximately 279 million metric tons of biomass residues, including bagasse and leaves (Chandel et al. 2012). Sugarcane bagasse (SCB), the fibrous material remaining after juice extraction, is produced in vast quantities as a byproduct of the sugar industry. Composed mainly of cellulose, hemicellulose, and lignin, it represents a valuable lignocellulosic biomass. Although a portion is reused within sugar and alcohol mills, about 30% remains underutilized (Michel et al. 2013).

Traditionally, bagasse was employed as a low‐cost boiler fuel or disposed of through open burning, practices that contribute to environmental pollution (Li et al. 2018; Wang et al. 2022). Early scientific efforts primarily focused on its energy potential, particularly for bioethanol production via lignocellulosic bioconversion (Chandel et al. 2012). However, recent years have witnessed a paradigm shift toward sustainable valorization, with growing interest in transforming bagasse into high‐value bioproducts such as xylitol, organic acids, specialty enzymes, and single‐cell proteins. Technological advances now enable the extraction of purified cellulose, cellulose nanofibers, and extracellular polymeric substances, extending bagasse applications into fields such as bioplastics, pharmaceuticals, food packaging, and wastewater treatment (Debnath et al. 2024; Mahmud and Rahman 2021; Tagne et al. 2024). Notably, nanocellulose derived from bagasse has gained attention for its potential in biodegradable food films and biomedical materials.

Globally, several countries have successfully integrated bagasse into their renewable energy and bioeconomy strategies. Brazil, for instance, uses bagasse as a principal feedstock for ethanol and cogenerated electricity (Zambello et al. 2024). In Africa, Angola has demonstrated promising progress by utilizing its favorable climatic conditions for sugarcane cultivation to produce low‐emission biofuels and renewable power (Matias et al. 2024). Despite these advances, substantial research gaps remain, particularly in exploring the incorporation of bagasse‐derived ingredients into functional foods and evaluating their nutritional and health‐promoting effects. Moreover, the lack of comprehensive toxicological assessments and clear regulatory frameworks continues to hinder the commercialization of bagasse‐based food and nutraceutical products.

While the potential of sugarcane bagasse as a sustainable biorefinery feedstock is widely recognized, its industrial adoption remains constrained by interlinked challenges. These barriers are not due to a lack of applications but rather stem from limitations in pretreatment efficiency, high operational costs, and complex regulatory requirements. Although numerous studies have demonstrated that SCB can be transformed into value‐added products such as bioethanol, biogas, bioplastics, and dietary fibers, the associated pretreatment and conversion processes often demand considerable energy and chemical inputs, hindering scalability (Galbe and Wallberg 2019). Techno‐economic analyses further reveal that enzyme costs, feedstock logistics, and capital expenditures remain key obstacles to commercialization (Mandegari et al. 2017). Regulatory compliance also poses difficulties, particularly in food‐related applications that require stringent safety evaluations and adherence to international standards (Hiranobe et al. 2024). Therefore, a comprehensive synthesis of technological performance, economic feasibility, and policy alignment is crucial to identifying viable pathways for integrating SCB into sustainable bio‐based industries.

Nanocellulose, produced as cellulose nanofibrils (CNF) or cellulose nanocrystals (CNC), has emerged as a promising material for packaging due to its renewable origin and superior mechanical and barrier properties. However, despite its environmental appeal, large‐scale production faces major economic and energy challenges. Life cycle assessments show that certain pretreatment routes, such as carboxymethylation, substantially increase environmental burdens owing to high solvent and energy consumption (Arvidsson et al. 2015). Similarly, techno‐economic studies indicate that CNC production from wood pulp using sulfuric acid hydrolysis entails minimum selling prices of US $4.69–$4.89 per kg and global warming impacts of 11.18–11.39 kg CO_2_ eq per kg of CNC (Rajendran et al. 2023). Manufacturing costs remain variable, often reaching thousands of dollars per ton, depending on feedstock, pretreatment method, acid usage, solvent recovery efficiency, and production scale (Kaur et al. 2021). Consequently, when integrating nanocellulose into biopolymer packaging systems, it is essential to recognize these cost and energy trade‐offs rather than assuming that bio‐based automatically equates to lower impact. Future efforts should focus on low‐energy pretreatments, waste biomass utilization, efficient solvent recovery, and process scaling to enhance both environmental and economic performance.

In many developing regions, particularly Sub‐Saharan Africa, industrial‐scale valorization of bagasse is hampered by insufficient technological infrastructure and underdeveloped policy frameworks. Furthermore, the economic viability and environmental impacts of advanced processing methods like nanocellulose extraction and enzymatic hydrolysis are not well understood. To address these gaps, this review presents a comprehensive and multidisciplinary assessment of sugarcane bagasse, focusing on its applications in food systems as a functional ingredient, its health‐related potential, and its broader industrial roles. The review also evaluates the economic and environmental implications of bagasse utilization. Ultimately, it highlights how sugarcane bagasse can contribute to national development through job creation, sustainable agriculture, and public health, while supporting global efforts in sustainability, innovation, and climate change mitigation.

## 2 Methodology

**FIGURE 1:**
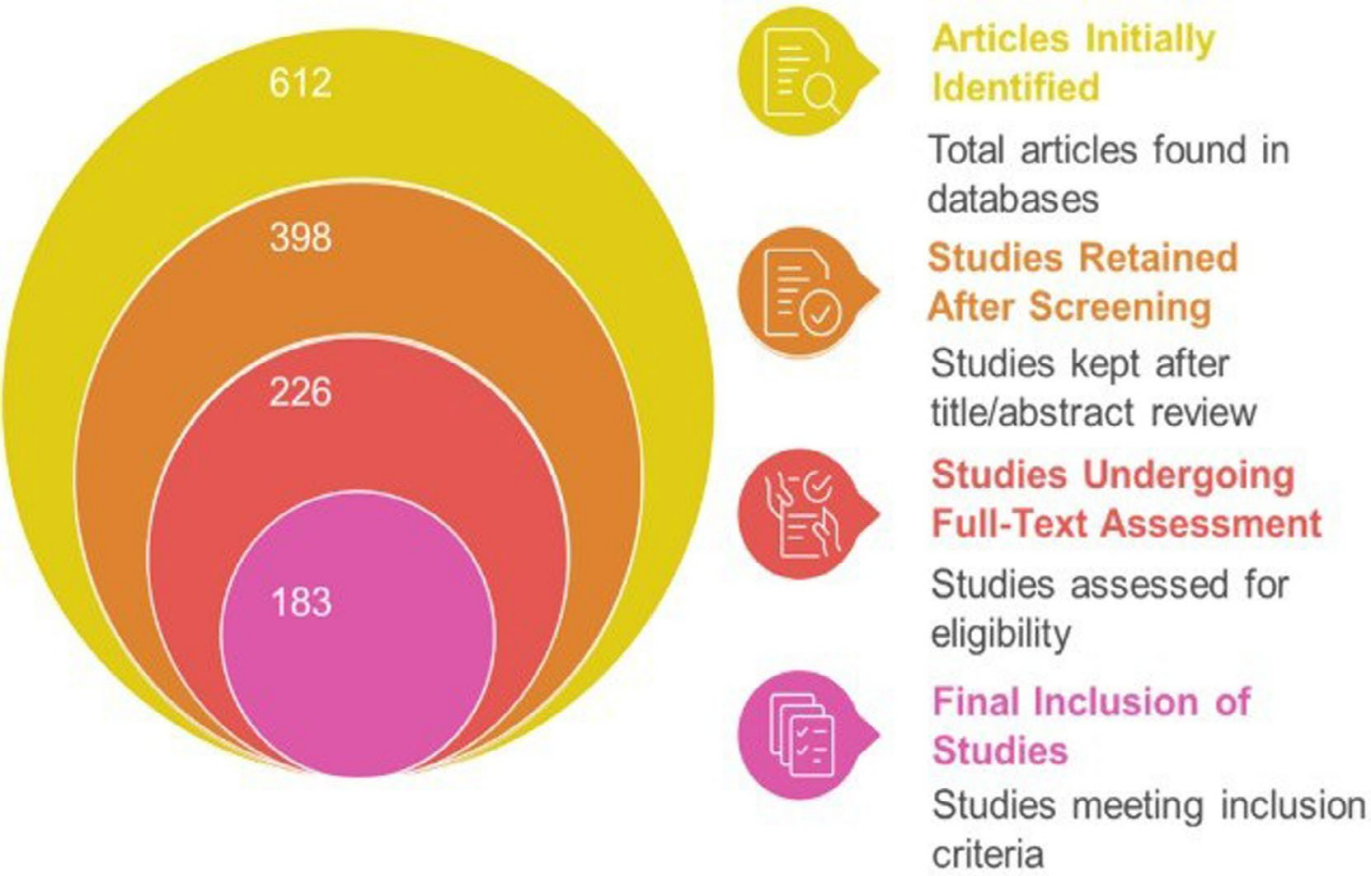
Literature review selection process performed in this review.

This narrative review employed a structured and transparent literature search to ensure rigor and reproducibility. Relevant publications were retrieved from major scientific databases, including Scopus, Web of Science, Science‐Direct, PubMed, and Google Scholar, covering studies published between 2000 and 2025, with emphasis on recent literature (2020–2025). A combination of general and specific keywords was used to capture relevant studies on sugarcane bagasse and lignocellulosic biomass. For example, a structured Boolean search string was applied as follows: (“sugarcane bagasse”) AND (“biorefinery” OR “valorization” OR “bio‐based products” OR “bioethanol production” OR “enzymatic hydrolysis” OR “dietary fiber” OR “functional foods” OR “nanocellulose” OR “circular bioeconomy” OR “health benefits”). This approach ensured comprehensive retrieval of studies on the valorization, bio‐based applications, and potential health impacts of sugarcane bagasse. A total of 612 articles were initially identified. After removing duplicates and conducting a preliminary screening, 398 studies were retained based on title and abstract relevance. Subsequently, 226 studies underwent full‐text assessment against the defined eligibility criteria, resulting in a final inclusion of 183 studies (Table 1 and Figure 1). Inclusion criteria comprised peer‐reviewed English‐language publications presenting experimental, analytical, or review data on sugarcane bagasse composition, conversion technologies, or applications in food, health, bioenergy, or industrial sectors. Exclusion criteria included non‐English papers, inaccessible full texts, conference abstracts, and studies unrelated to bagasse valorization (e.g., those limited to sugarcane juice or molasses). Data from the selected studies were extracted and categorized into five thematic areas: bioenergy production, biodegradable materials and packaging, food and feed applications, environmental remediation and soil fertility, and economic and policy implications. A manual thematic analysis was performed to identify recurring concepts, emerging technologies, and research gaps. In addition to recent works, earlier seminal studies were included selectively to provide historical and methodological context for contemporary research on sugarcane bagasse valorization.

**TABLE 1:** Summary of literature screening and inclusion process.

Stage	Description	Number of articles
Initial database search	Records identified from Scopus, Web of Science, Science Direct, PubMed, and Google Scholar	612
Duplicate removal and abstract screening	Excluded non‐relevant and duplicate records	398
Full‐text assessment	Articles were evaluated for detailed eligibility	226
Final inclusion	Studies meeting all inclusion criteria	183

## 3 Applications of Sugarcane Bagasse

### 3.1 Bioenergy Production

**FIGURE 2:**
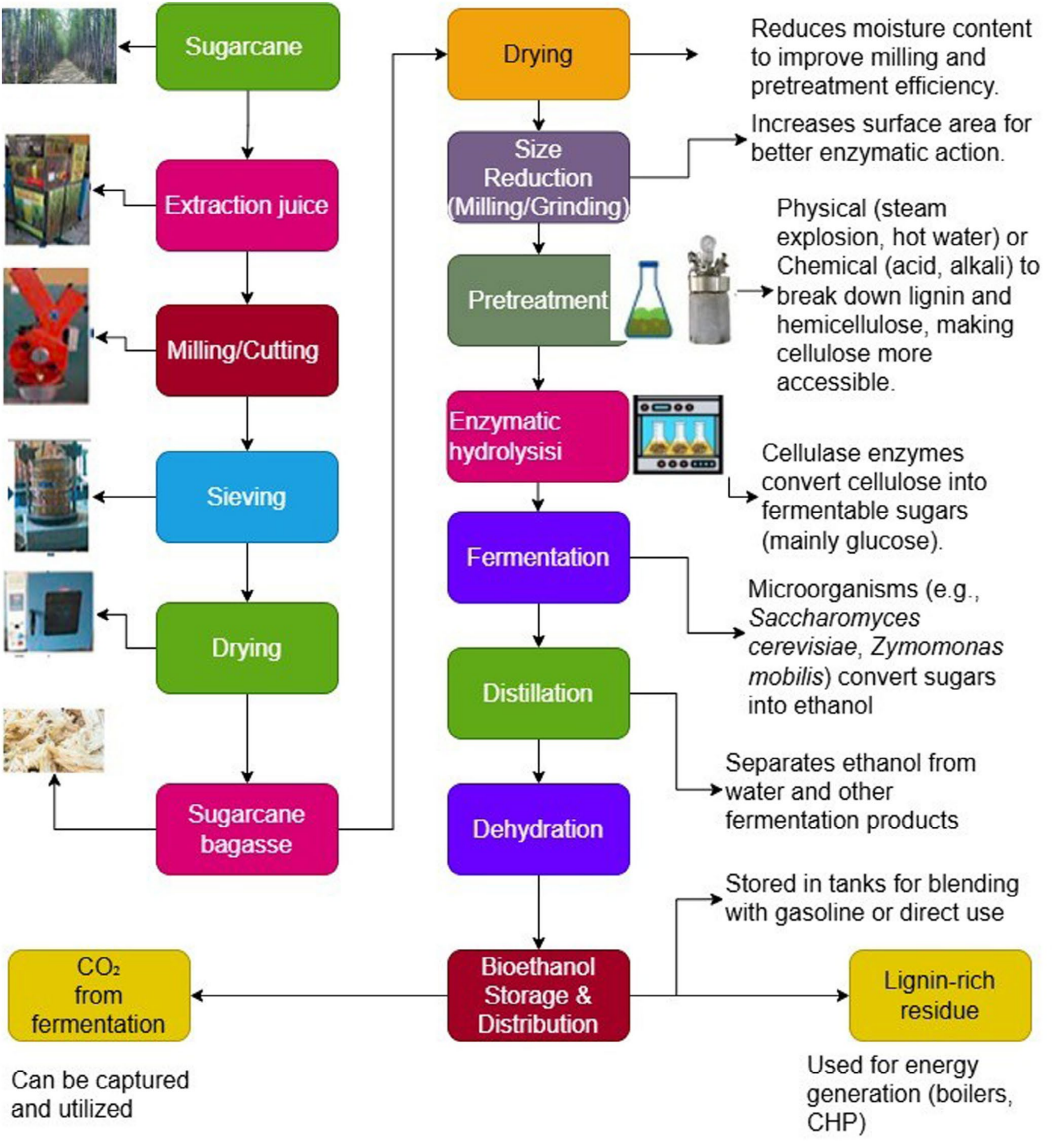
Simplified process flow diagram for bioethanol production from sugarcane bagasse, including pretreatment, enzymatic hydrolysis, fermentation, and product recovery steps.

Sugarcane bagasse, the fibrous lignocellulosic residue generated after juice extraction (Figure 2), is one of the most abundant agro‐industrial byproducts worldwide, with an estimated annual global production of approximately 279 million metric tons (Singh et al. 2024). Traditionally, bagasse has been combusted within sugar mills to produce steam and electricity, primarily to meet on‐site energy demands and occasionally to export surplus power to the grid. While this conventional use remains significant, large volumes of wet bagasse continue to be stockpiled or openly burned, causing environmental pollution and fire hazards (Zafeer et al. 2024).

In recent years, both research and industrial efforts have increasingly focused on advanced bioenergy pathways that valorize sugarcane bagasse through biochemical and thermochemical conversion technologies. These approaches enable the production of a wide range of renewable energy products, including bioethanol, biomethane, biobutanol, biohydrogen, biochar, biopellets, and syngas. Among these, bioethanol production remains the most extensively studied route, primarily due to bagasse's high cellulose content (~50%), which supports efficient conversion when effective pretreatments remove lignin and enhance enzymatic accessibility (Chen et al. 2012; da Silva Delabona et al. 2012).

Pretreatment method and severity play a critical role in conversion efficiency. Lignocellulosic bagasse requires effective delignification and hydrolysis; incomplete pretreatment leaves cellulose and hemicellulose inaccessible, thereby reducing fermentable sugar concentrations. Alkali pretreatment, particularly with 10% NaOH, has been widely reported to enhance delignification and enzymatic hydrolysis, achieving saccharification efficiencies as high as 489.5 mg/g and outperforming acid‐based methods (Tiwari et al. 2022; Wunna et al. 2021). Consequently, higher glucan availability from alkali‐pretreated bagasse often translates into improved ethanol yields.

Under optimized fermentation conditions, *Saccharomyces cerevisiae* strains have produced ethanol concentrations up to 3.7% v/v with commercial strains and 3.3% v/v with local isolates (Baz et al. 2017). Similarly, studies using hydrolyzed bagasse have achieved around 3.1% v/v ethanol, with alkali pretreatment again identified as a key factor enhancing cellulose accessibility (Restiawaty et al. 2020; Triyani et al. 2023). However, much higher ethanol concentrations have been demonstrated in other optimized systems: for instance, a recent study employing whole sugarcane and simultaneous saccharification fermentation achieved 86.7 g/L ethanol (~11.0% v/v), highlighting the effect of higher sugar loading, aggressive pretreatment, and process optimization (van Dyk et al. 2025).

Yeast strain physiology is another major determinant of ethanol yield and tolerance. Commercial or industrial *S. cerevisiae* strains are often engineered or selected for higher ethanol productivity, osmotic resistance, and ethanol tolerance, typically achieving > 8%–10% v/v ethanol under industrial conditions (da Silva Fernandes et al. 2022). In contrast, local or native strains may show lower productivity due to limited stress resistance, explaining the variability in ethanol concentrations across studies.

**FIGURE 3:**
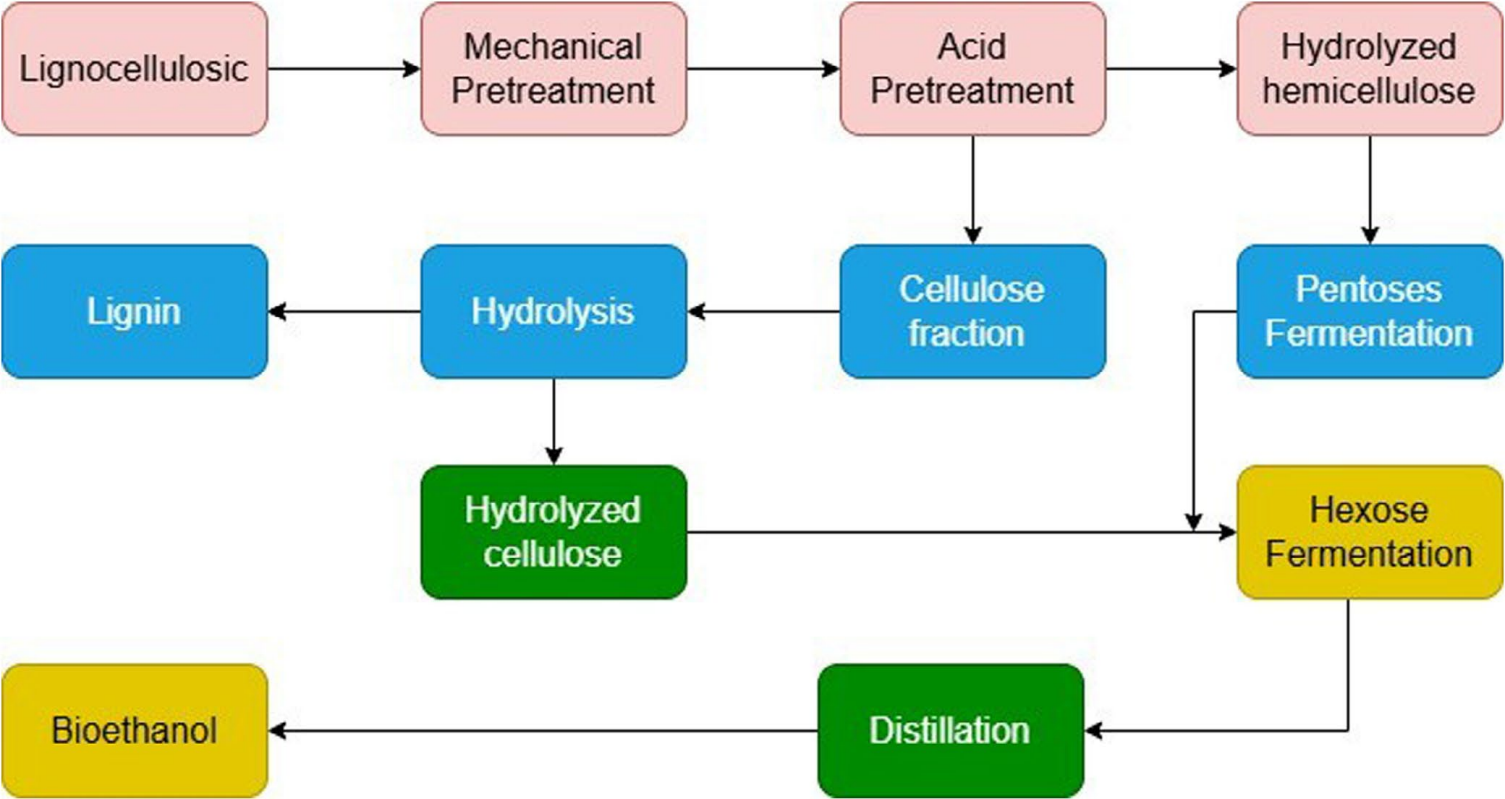
A flowchart for bioethanol production from lignocellulosic biomass waste.

Beyond ethanol, lignocellulosic bioconversion pathways from bagasse can also yield value‐added co‐products such as xylitol, organic acids, specialty enzymes, and single‐cell proteins (Figure 3) (Chandel et al. 2012; Li et al. 2018). Together, these integrated biorefinery strategies enhance the overall economic and environmental value of bagasse utilization.

Anaerobic digestion represents another promising bioenergy avenue, producing biomethane yields up to 347.6 mL CH_4_/g volatile solids (Agarwal et al. 2022). Pretreatment methods such as steam explosion and hydrothermal processing have been shown to increase gas yields and purity. Additionally, co‐digestion strategies, like blending sugarcane bagasse with fruit and vegetable wastes in a 5:95 ratio, have enhanced methane production by 27.6% compared to mono‐digestion (Alino et al. 2022). The nutrient‐rich digestate resulting from anaerobic digestion can be further processed into biochar, thus creating a dual benefit of renewable energy generation and improved soil fertility (Agarwal et al. 2023).

**FIGURE 4:**
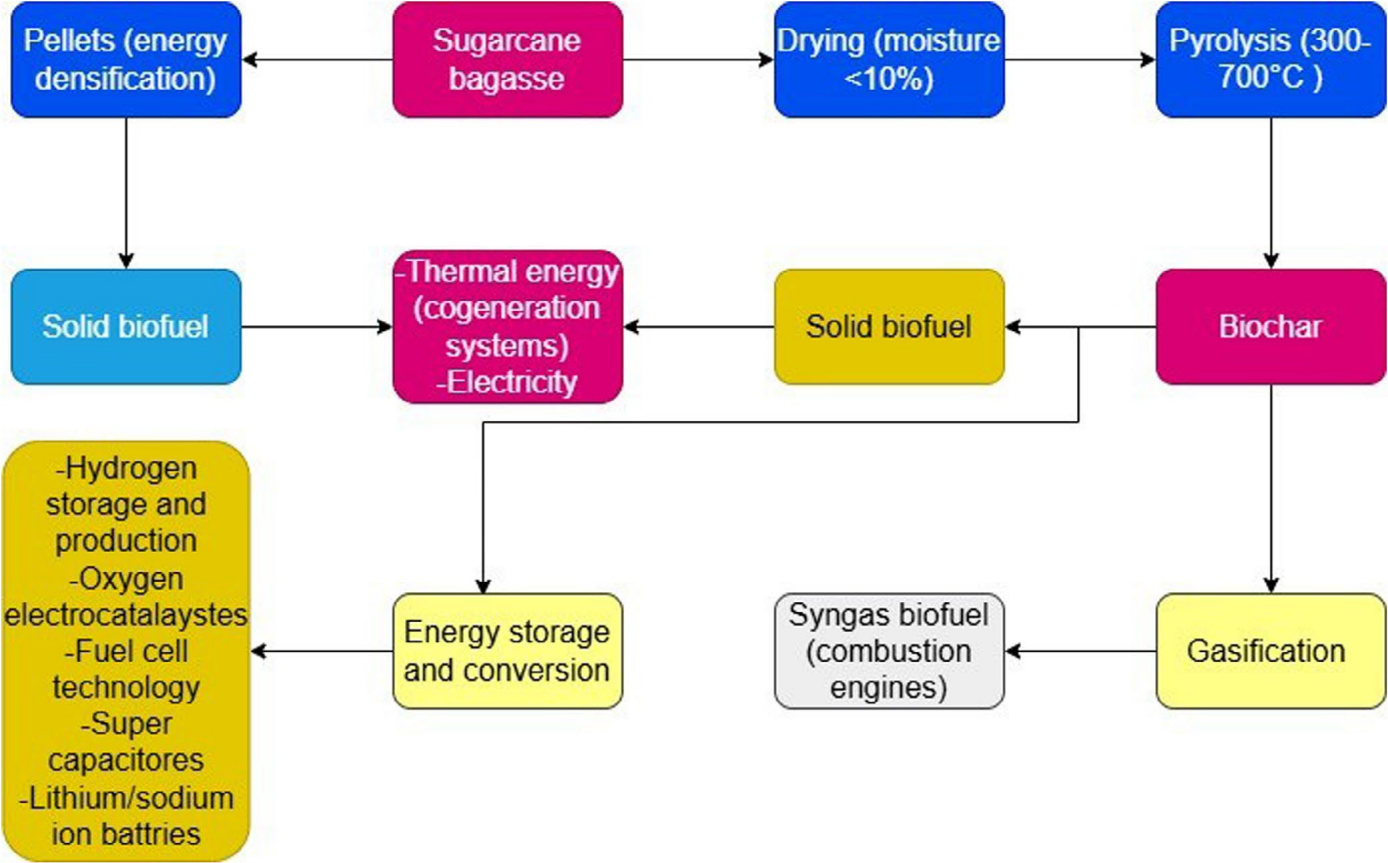
A flowchart illustrating two pathways for energy utilization from sugarcane bagasse.

Thermochemical conversion technologies, including pyrolysis, gasification, and hydrothermal carbonization (HTC), further expand the bioenergy potential of sugarcane bagasse (Figure 4). Pyrolysis conducted at 300°C–600°C produces biochar characterized by a high surface area (up to 198 m^2^/g), rich in stable carbon and essential nutrients, making it highly effective for soil amendment, carbon sequestration, and as a precursor to activated carbon (Ahmad et al. 2014; Jamilatun et al. 2022). Gasification converts bagasse into syngas, a combustible mixture mainly composed of carbon monoxide and hydrogen usable for power generation or further processed into hydrogen for energy storage applications (Aktawan and Salamah 2018; Deng et al. 2023). HTC, operating at relatively mild temperatures of 180°C–260°C under pressure, transforms wet bagasse into hydrochar, which shows potential in environmental remediation, energy storage devices, and soil improvement (Hoekman et al. 2013; Khan et al. 2021).

Additionally, solid biofuels such as biopellets and briquettes produced through densification techniques improve the energy density, handling, and transport efficiency of bagasse. These fuels are suitable for biomass boilers and industrial furnaces and provide a cleaner alternative to coal, contributing to reduced emissions (Gunjal 2021; Kaliyan and Morey 2009; Zhang et al. 2024). Given the substantial global production of bagasse, the resource potential for biopellets and other solid biofuels is considerable (Singh et al. 2024).

Beyond energy production, bagasse supports various biotechnological applications. Microorganisms, including fungi and bacteria, produce enzymatic cocktails that hydrolyze biomass into valuable chemicals. Advances in genetic engineering and microbial strain development have further improved these processes' efficiency (Antoniêto et al. 2022). Enzymes from *Trichoderma* species, for example, have been found effective in enhancing the digestibility of sugarcane silage, increasing xylooligosaccharide production, and supporting bio‐bleaching of cellulose (Copete‐Pertuz et al. 2019; da Costa et al. 2019). Optimal bioethanol production depends on precise control of fermentation parameters such as temperature and humidity, underscoring the importance of process optimization for maximizing yields (Mizar et al. 2020).

The environmental benefits of bagasse‐derived bioenergy are notable. Combustion of bioethanol produces lower greenhouse gas emissions compared to fossil fuels, contributing to improved air quality and soil health (Mizar et al. 2020). Gasification provides a cost‐effective source of syngas predominantly composed of carbon monoxide (Aktawan and Salamah 2018). Moreover, biochar, generated through oxygen‐free thermal decomposition of biomass, possesses high porosity, large surface area, and stability, which facilitate diverse applications ranging from soil amendment to carbon sequestration and environmental remediation (Bashir et al. 2020; Jacob et al. 2022; Khan et al. 2020; Monisha et al. 2022).

**FIGURE 5:**
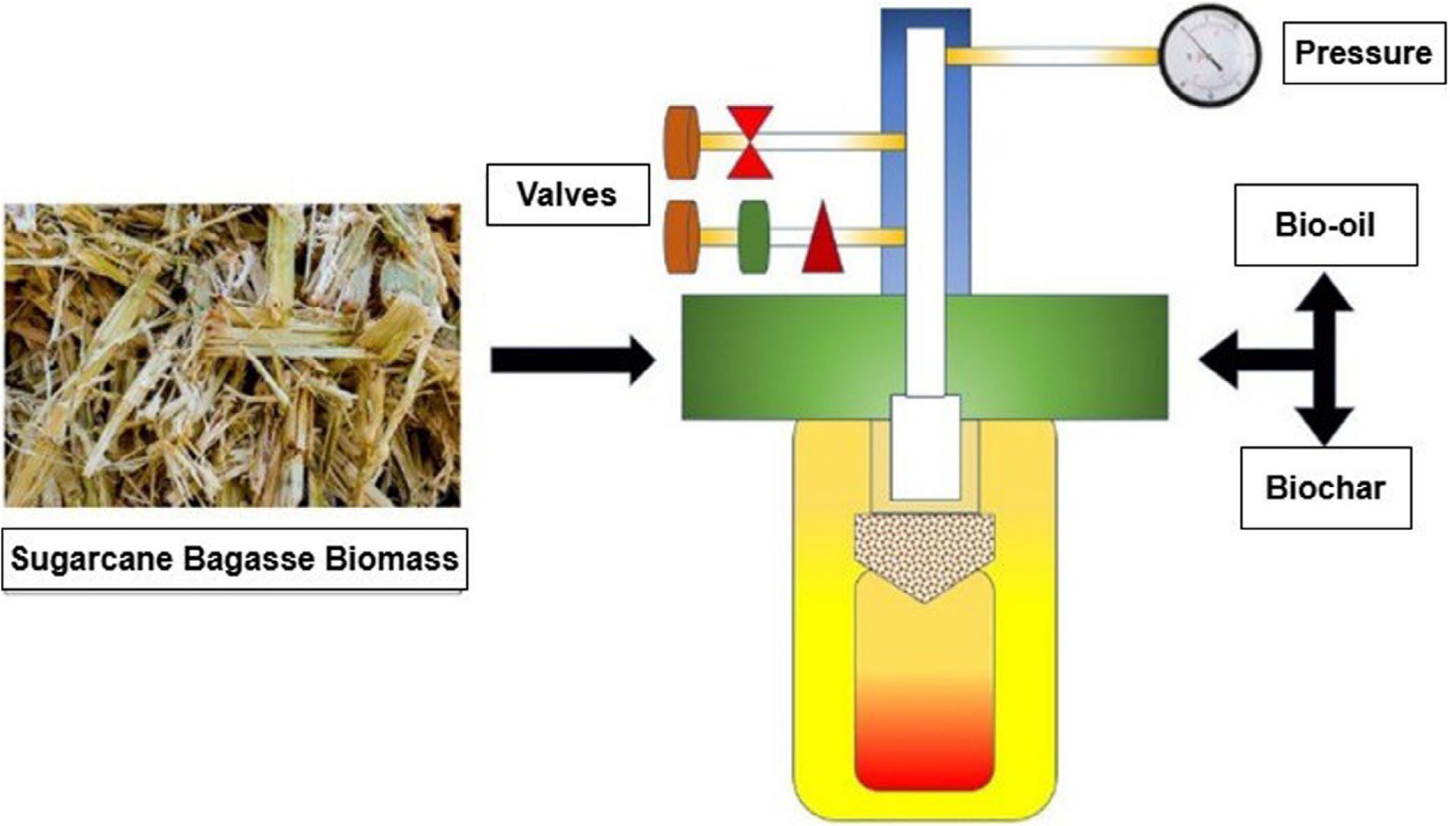
A typical hydrothermal carbonization reactor is used for converting sugarcane bagasse biomass into biochar.

**FIGURE 6:**
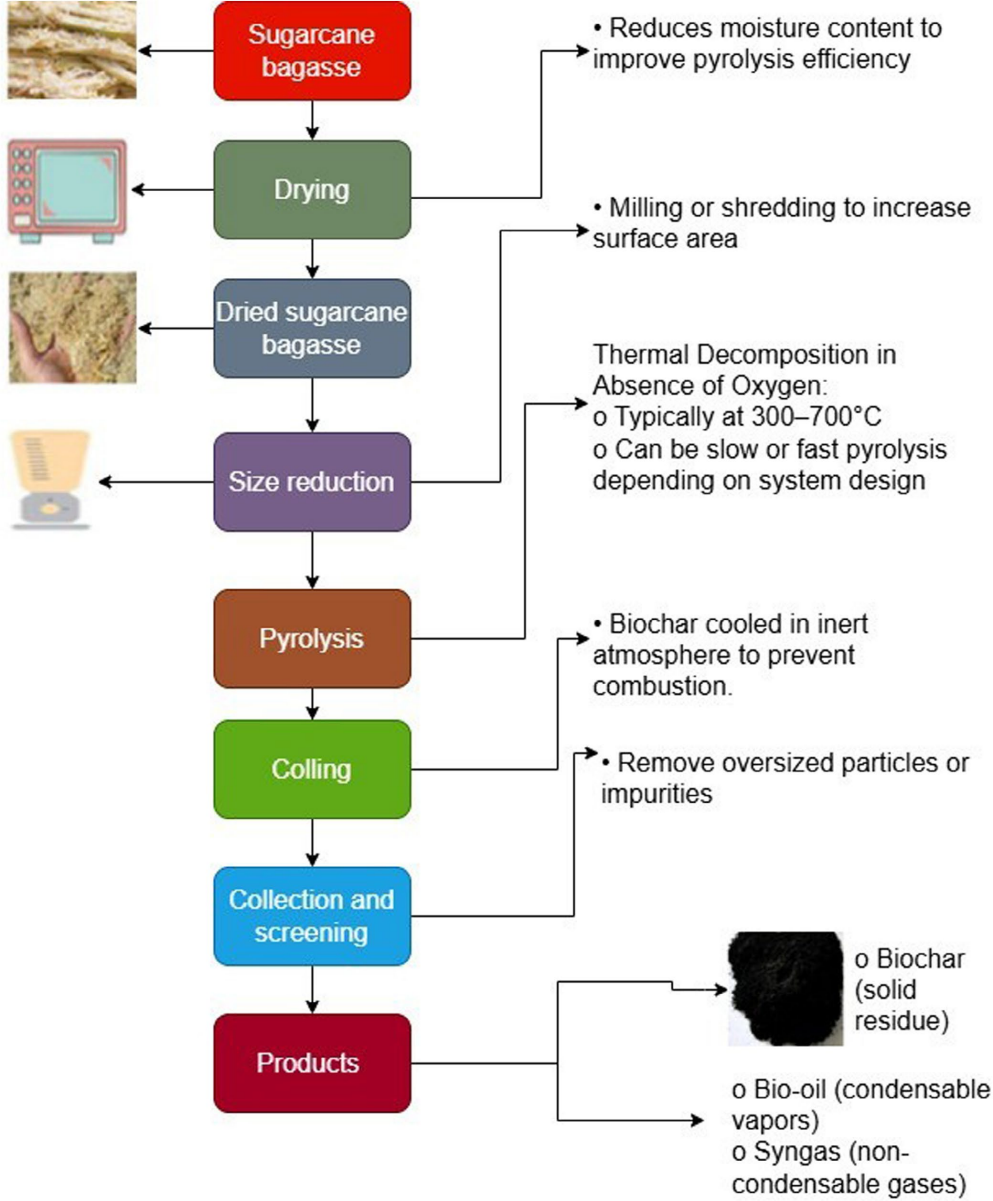
Simplified process flow diagram for biochar production from sugarcane bagasse via pyrolysis, including drying, thermal decomposition, and product recovery.

Figures 4 and 6 illustrate key bioenergy pathways from sugarcane bagasse. One pathway produces pellets as a solid biofuel for thermal and electrical energy generation. Another pathway involves producing biochar through pyrolysis, which can be further utilized for syngas production to power combustion engines or serve as a solid biofuel (de Almeida et al. 2022; Iwuozor et al. 2022). A typical hydrothermal carbonization reactor used for biochar and bio‐oil production is shown in Figure 5. Biochar's applications extend to energy conversion and storage, including hydrogen production and storage, supercapacitors, and lithium/sodium‐ion batteries (Deng et al. 2023; Emenike et al. 2024; Hsiao et al. 2023; Sang et al. 2024).

### 3.2 Pretreatments for Enhanced Bioethanol Production

Various pretreatment methods have been developed to enhance the conversion efficiency of sugarcane bagasse (SCB) into fermentable sugars for bioethanol production, and each method differs in its target components, advantages, and limitations. Compared to other techniques, acid pretreatment is one of the most commonly used because it effectively hydrolyzes hemicellulose, thereby improving enzyme accessibility to cellulose and increasing ethanol yield (Johannes and Xuan 2024). However, despite its high efficiency, acid pretreatment requires substantial energy and chemical input and tends to produce inhibitory compounds such as furfural and hydroxymethylfurfural (HMF), which can hinder fermentation unless detoxification is applied (Table 2). In contrast, alkali pretreatment focuses mainly on lignin removal, which greatly enhances cellulose accessibility and fermentable sugar recovery (Johannes and Xuan 2024). While this method generally results in higher enzymatic hydrolysis rates and ethanol yields than acid pretreatment, it also consumes more water, requires longer processing times, and demands costly chemical recovery, making it less economical for large‐scale operations.

**TABLE 2:** Comparison of common pretreatment methods for sugarcane bagasse and microalgal biomass.

Pretreatment method	Target component	Advantages	Disadvantages	Impact on bioethanol production	References
Acid pretreatment	Hemicellulose (Sugarcane Bagasse, *Chlorella vulgaris* )	Effective solubilization of hemicellulose enhances enzymatic hydrolysis; increases sugar availability for fermentation.	Generates inhibitory compounds such as furfural and hydroxymethylfurfural (HMF); requires high chemical and energy input; needs neutralization and detoxification steps.	Significantly increases sugar release and ethanol yield if detoxification is properly managed. Excess inhibitors can reduce fermentation efficiency.	Johannes and Xuan (2024), Mankar et al. (2021), and Damayanti et al. (2025)
Alkali pretreatment	Lignin (Sugarcane Bagasse)	Efficient lignin removal improves cellulose accessibility, enhances fermentable sugar recovery, and enzymatic hydrolysis rate.	High water consumption, long pretreatment duration, and a costly chemical recovery process.	Leads to higher ethanol yield and better enzymatic efficiency compared to untreated bagasse.	Johannes and Xuan (2024) and Prasanthan et al. (2025)
Steam explosion	Hemicellulose and partial lignin	Rapid processing requires minimal chemicals; it increases cellulose surface area and accessibility.	Needs specialized high‐pressure equipment; risk of sugar degradation and inhibitor formation at high temperatures.	Produces high sugar yield and improved ethanol output when optimized for temperature and retention time.	Barciela et al. (2023) and Mankar et al. (2021)
Ionic liquid/deep eutectic solvent (des) pretreatment	Lignin and Hemicellulose	High delignification efficiency; preserves cellulose structure; environmentally friendly and recyclable.	High solvent cost; challenges in solvent recovery and scale‐up.	Improves enzymatic digestibility and bioethanol yield, but economic feasibility requires optimization.	Mankar et al. (2021)
Biological pretreatment	Lignin	Uses lignin‐degrading fungi; environmentally friendly and low‐energy method; minimal inhibitor formation.	Requires long processing times; limited scalability; risk of incomplete delignification.	Enhances downstream enzymatic hydrolysis; suitable for eco‐friendly operations but slow for industrial use.	Mankar et al. (2021)
Ultrasonic pretreatment (microalgal context)	Cell wall polysaccharides (Spirulina)	Effective disruption of microalgal cell walls improves sugar release during hydrolysis.	High energy demand; less suitable for large‐scale lignocellulosic systems.	Enhances fermentable sugar yield in microalgae‐based bioethanol production.	Damayanti et al. (2025)
Thermal acid pretreatment (microalgal context)	Cell wall polysaccharides (Chlorella)	Most effective for breaking tough Chlorella cell walls; increases reducing sugar yield.	May cause degradation of sensitive compounds if not controlled. Risk of phenolic inhibitor formation	Produces high sugar concentrations and bioethanol yield in microalgal systems.	Damayanti et al. (2025)

Similarly, steam explosion offers another approach, but it differs by combining physical and chemical actions. This method involves exposing SCB to high‐pressure steam followed by sudden decompression, which disrupts the fiber structure and increases cellulose accessibility (Barciela et al. 2023) (Table 2). Unlike acid or alkali pretreatments, steam explosion minimizes chemical use and allows faster processing. Nevertheless, it requires specialized high‐pressure equipment, and excessive temperature or retention time can degrade sugars and generate inhibitors.

Overall, while acid pretreatment is more effective for rapid hemicellulose hydrolysis, alkali pretreatment performs better in lignin removal, and steam explosion provides a balance between efficiency, cost, and environmental sustainability. Therefore, the selection of the most suitable method depends on the intended application, available resources, and economic feasibility. Increasingly, researchers are turning toward hybrid or integrated pretreatment systems that combine the strengths of multiple methods to achieve higher conversion efficiency and better overall performance in bioethanol production.

### 3.3 Sugarcane Bagasse Utilization in Biodegradable Products and Sustainable Packaging

**FIGURE 7:**
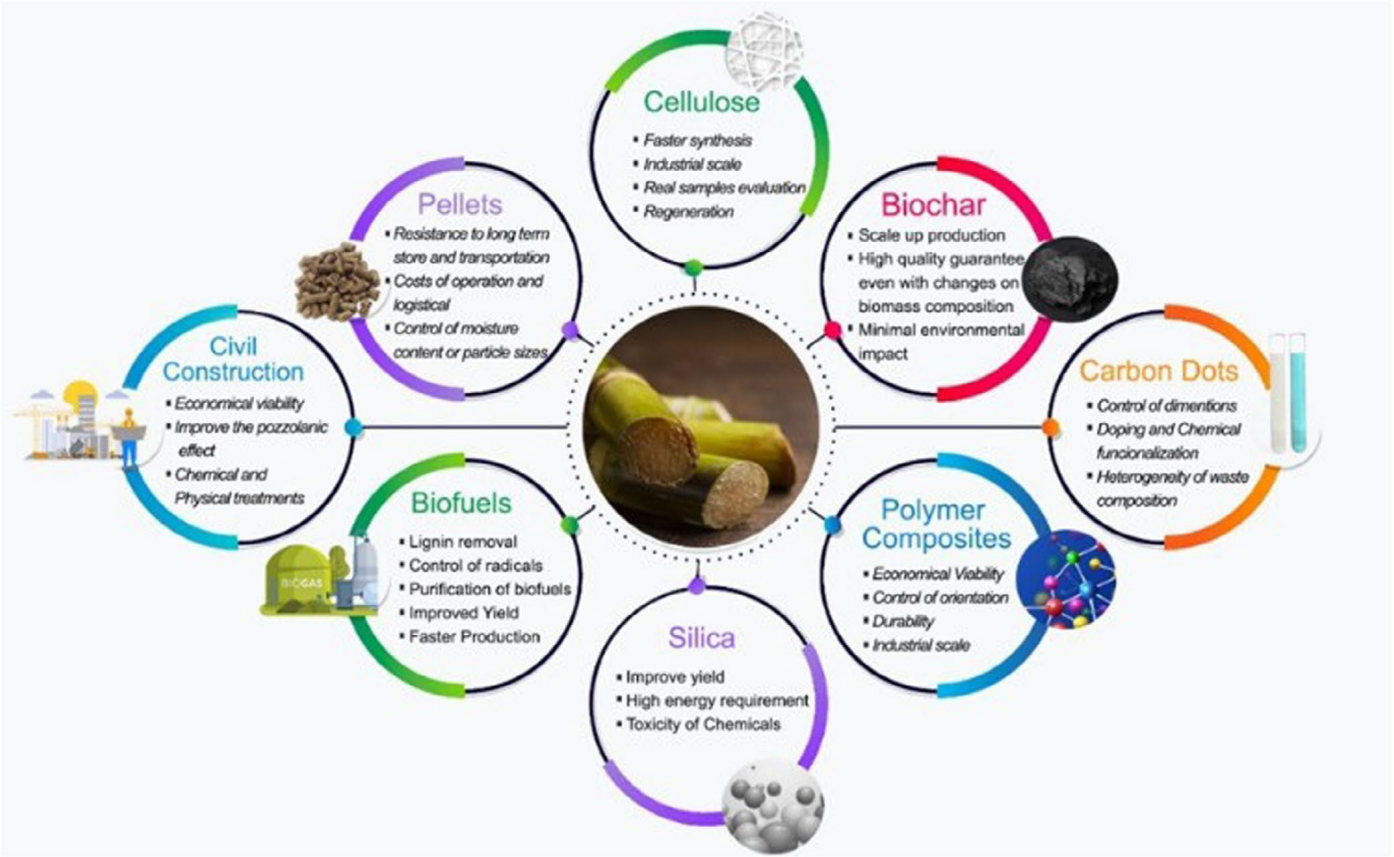
Summarized applications of sugarcane bagasse and challenges of each field.

The environmental impact of conventional plastic products has driven the exploration of biodegradable alternatives, such as bagasse‐based products. Biodegradable plates made from sugarcane waste offer an eco‐friendly, safe, and cost‐effective alternative to traditional plastic plates (Hossam and Fahim 2023). Sugarcane bagasse provides an excellent alternative for producing biodegradable products such as plates, cups, and various packaging materials. This fibrous residue from sugarcane juice extraction offers a key advantage over traditional plastics due to its biodegradability and compostability. Bagasse‐based products are also versatile and practical, as they can be safely stored in freezers and used in microwaves (Wang et al. 2022). The valorization of sugarcane bagasse for food packaging involves utilizing cellulose fibers from sugarcane processing to produce sustainable packaging materials (Figure 7). Research efforts have focused on improving these materials through chemical modification, blending, and nano‐fibrillation. Sugarcane bagasse‐based packaging provides excellent mechanical strength, barrier properties, biodegradability, and a reduced environmental impact compared to conventional plastics, making it a promising alternative in packaging technology (Janika et al. 2024).

Besides being used as low‐cost fuel, bagasse's rich cellulose, hemicellulose, and lignin content make it a versatile material for biodegradable packaging, thus helping to manage waste and reduce landfill burdens. Chemical modifications and blending with other biopolymers improve the properties of bagasse‐based packaging, addressing issues like mechanical strength and barrier properties. Innovations such as incorporating natural preservatives into biodegradable materials further extend their applications in food packaging.

For wider adoption of biodegradable products, partnerships with governments, innovative funding models, and public awareness campaigns are essential. Educating consumers about the benefits of biodegradable alternatives and supporting policies that incentivize their use can drive a significant shift toward sustainability. By focusing on scalability, accessibility, and continuous innovation, the shift toward bagasse‐based products can effectively contribute to reducing plastic waste and promoting a greener future (Abdou et al. 2022; Antoniêto et al. 2022).

### 3.4 Physicochemical Properties and Food Applications of Sugarcane Bagasse

#### 3.4.1 Composition and Functional Characteristics

Sugarcane bagasse (SCB), the fibrous residue remaining after juice extraction from sugarcane stalks, is rich in dietary fiber, mainly cellulose (40%–50%), hemicellulose (30%–35%), and lignin (10%–14%) (Dimopoulou and Kontogiorgos 2020). This composition makes SCB a valuable source of dietary fiber rather than macronutrients. Its functional properties, such as high water‐holding and oil‐binding capacity, enhance dough hydration, texture, and shelf life (Avendaño‐Rito et al. 2024; Vijerathna et al. 2019; Nguyễn et al. 2022). Moreover, SCB incorporation reduces caloric density while improving dietary fiber, protein, and antioxidant levels (Lamas and Gende 2023; Romero‐Lopez et al. 2011; Silva et al. 2020; Tombini et al. 2024).

Image analysis techniques have been applied to assess SCB effects on color and porosity in baked goods, helping to optimize product quality (Darapoor et al. 2023). Moderate additions (5%–15%) are generally acceptable to consumers and enhance nutritional and sensory properties (Tombini et al. 2024; Vijerathna et al. 2019; Nguyễn et al. 2022). Additionally, sugarcane bagasse's functional properties, such as high oil and water holding capacities, contribute to its effectiveness as a sustainable ingredient in food production (Leang and Saw 2011).

#### 3.4.2 Pretreatments to Enhance the Functional Properties of Bagasse Products

Sugarcane bagasse can undergo various chemical treatments to enhance its functional properties. For example, treating samples with dichromate solution reduces the crystallinity index, signifying effective removal of polar impurities. The raw fibers have a crystallinity index of 72%, while those treated with permanganate and dichromate show indices of 47% and 50%, respectively (Prabhu et al. 2022). Additionally, pretreatment with aqueous acetic acid followed by post‐treatment can improve enzymatic polysaccharide conversion and cellulose digestibility (Bai et al. 2023).

Different treatments, such as microwave‐assisted alkaline treatments, have effectively removed lignin, making bagasse suitable for use in nutraceutical products like chapatti‐type bread and noodle‐type pasta (Gil‐López et al. 2019a).

Furthermore, bagasse's dietary fibers, when subjected to chemical treatments, show promising improvements in their physicochemical and functional properties, such as water‐holding capacity and oil‐binding capacity (Allam et al. 2024). Allam et al. (2024) highlight sugarcane bagasse as a valuable source of dietary fiber and functional ingredients. NaOH+PAA (Peracetic acid) treatment yielded fibers with the lowest lignin content (1.65%), highest holocellulose content (93.07%), and highest whiteness index (83.37). These fibers also had a high crystallinity index and improved water‐holding capacity (WHC) and oil‐binding capacity (OBC). Reducing the particle size of treated samples decreased WHC and OBC but increased α‐amylase inhibitory activity. NaOH+PAA is a promising method for extracting fibers from sugarcane bagasse under moderate conditions (Allam et al. 2024). Allam et al. (2024) highlight the potential of sugarcane bagasse as a valuable source of dietary fiber and functional ingredients. These authors found that NaOH + PAA treatment effectively extracted fibers with the lowest lignin content (1.65%), highest holocellulose content (93.07%), and highest whiteness index (83.37%). These fibers exhibited a high crystallinity index, enhanced water‐holding capacity (WHC), and improved oil‐binding capacity (OBC). However, reducing the particle size of treated samples decreased WHC and OBC while increasing α‐amylase inhibitory activity. According to these findings, NaOH+PAA is a promising method for extracting high‐quality fibers from sugarcane bagasse under moderate conditions.

Paroha (2020) investigated the effects of various treatments on bagasse, including steam, and acid or alkali at concentrations of 0.1, 0.25, 0.5, 0.75, and 1 N, applied for 15, 30, 45, and 60 min. These authors assessed functional properties such as solubility index (1%–3.25%), swelling power (0.6%–1.03%), water absorption capacity (6.8–9.7 g/g), and oil absorption capacity (2.47–9.10 g/g). The authors claimed that the treated bagasse performed significantly better than imported commercial products, demonstrating enhanced functional properties.

#### 3.4.3 Applications in Baked and Processed Foods

Recent formulation studies using sugarcane bagasse (SCB) or SCB‐derived fiber in bakery and staple foods show that low to moderate inclusion levels (≈3%–15%) significantly increase total dietary fiber (TDF) while maintaining good sensory quality. However, higher substitutions (> 15%–20%) often lead to undesirable texture or flavor changes unless processing adjustments or additives (such as emulsifiers or hydrocolloids) are used. Different food products and their maximum or acceptable incorporation levels are presented in Table 4.

##### 3.4.3.1 Biscuits

Sugarcane bagasse has shown great potential as an ingredient in biscuit production, enhancing both nutritional value and sustainability. Bagasse flour, which contains approximately 30.62% carbohydrates, 1.71% protein, 49.51% fiber, and 0.29% fat, serves as an excellent source of dietary fiber. Incorporating bagasse flour into biscuit formulations significantly increases fiber content while reducing carbohydrates, protein, and fat levels. For instance, biscuits enriched with 20% bagasse flour exhibited a 32.39% increase in fiber, accompanied by reductions of 11.82% in carbohydrates, 8.9% in protein, and 25.73% in fat compared with standard formulations (Firjatu et al. 2022). The high fiber content of these biscuits contributes to digestive health and helps alleviate constipation.

Further studies have demonstrated that substituting wheat flour with peeled sugarcane bagasse powder at levels of 5%, 10%, and 15% effectively enhances the fiber and ash contents of biscuits while maintaining desirable sensory properties (Nguyễn et al. 2022). Similarly, Ojha and Verma (2023) reported that incorporating up to 15% bagasse powder improved the nutritional profile and sensory acceptability of biscuits, confirming that such inclusion levels are well tolerated by consumers. Nguyễn et al. (2022) also found that biscuits containing 5%–15% peeled bagasse were classified as high‐fiber foods with comparable overall acceptability to control samples. Although the addition of bagasse slightly increased biscuit hardness and brightness, it did not significantly affect their diameter or thickness.

In addition, the use of steamed sugarcane bagasse has been found to enhance its safety and nutritional value by increasing hemicellulose B content. When 10% of wheat flour was replaced with steamed bagasse flour in biscuit formulations, the resulting product maintained proximate composition and quality comparable to that of control biscuits (Sangeetha et al. 2011). These fiber‐rich biscuits are particularly suitable for individuals with constipation, obesity, or colon‐related health conditions.

Overall, the incorporation of sugarcane bagasse, especially the peeled and steamed varieties, demonstrates its feasibility as a valuable functional ingredient in biscuit production. Utilizing this agro‐industrial by‐product not only promotes sustainable food processing but also supports the development of healthier, fiber‐enriched snack options.

##### 3.4.3.2 Bread

In bread production, sugarcane bagasse has been used to increase dietary fiber content, beneficial for digestive health. Adding bagasse can affect dough and bread properties, such as volume and firmness. For example, adding steamed sugarcane bagasse at a 10% replacement for wheat flour yields high‐fiber bread with acceptable rheological, nutritional, and sensory characteristics (Sangeetha et al. 2011).

Furthermore, the addition of sucrose ester to bread dough can improve dough expansion and bread properties, though higher dietary fiber levels may negatively impact sensory attributes (Sangnark and Noomhorm 2004). The addition of sugarcane bagasse (SCB) at levels ranging from 0% to 10% can lead to a substantial improvement in its nutritional value, while also enhancing its physical and sensory properties of bread (Orain Porter 2022). Sugarcane bagasse can be successfully incorporated into bread at low substitution levels (5%) without significantly compromising the quality, particularly when fine particles are used (Sangnark and Noomhorm 2003). SCB fibers treated with NaOH and assisted by microwave irradiation were added to chapatti‐type fermented bread at different percentages (up to 20%). The best results were obtained with 8% SCB, which showed improvements in the nutritional value of the bread. The addition of SCB increased the total dietary fiber content of the bread from 7.4 to 11.7 g/100 g. Bread with 8% SCB also showed increased antioxidant activity compared to control bread, indicating that adding SCB fibers may offer additional health benefits, such as enhanced antioxidant properties and lower glycemic index (Gil‐López et al. 2019b).

##### 3.4.3.3 Cookies

Sugarcane bagasse has been explored as an ingredient in cookie production, enhancing the fiber content and nutritional profile. Incorporating 5% bagasse (with peel) into cookies has been found viable for commercial production, maintaining acceptable sensory characteristics while adding nutritional value (Vijerathna et al. 2019). Additionally, cookies with 20% bagasse flour show increased fiber content but may become harder and less preferred compared to controls (Licona‐Aguilar et al. 2023). The impact of bagasse on cookie texture and sensory qualities underscores the need to balance health benefits with consumer acceptability. Adding up to 5% bagasse powder was found to improve the fiber content without significantly affecting sensory characteristics. However, higher levels of bagasse led to changes in color and texture, and a balance between nutritional benefits and consumer acceptability is crucial (Nguyễn et al. 2022). Similarly, cookies with up to 10% bagasse have shown positive sensory qualities while increasing the fiber content significantly (Nguyễn et al. 2022). As the amount of sugarcane bagasse (SCB) in cookie formulations increases from 10% to 30%, there is a significant rise in the cookies' dietary fiber content.

For instance, cookies with 30% SCB contain nearly 11% dietary fiber, compared to less than 1% in the control sample. Despite this addition, the study revealed that SCB had little effect on the cookies' moisture content, which is crucial for maintaining their texture and shelf life. However, the inclusion of SCB slightly altered the ash and protein levels, likely due to its mineral content. The fibrous nature of the bagasse also contributed to increased hardness in cookies with higher SCB concentrations. Nevertheless, sensory evaluations showed that even cookies with up to 30% SCB were well received, particularly in terms of flavor, aroma, and texture (Morales‐Tapia et al. 2023). Furthermore, the addition of bagasse powder into cookies has been investigated, with 5% bagasse‐enriched cookies exhibiting high sensory acceptability and improved fiber content (Vijerathna et al. 2019).

##### 3.4.3.4 Cakes and Donuts

Incorporating sugarcane bagasse into cakes, such as oat flour and banana cakes, improves texture and increases dietary fiber content. The addition of bagasse results in firmer cakes with enhanced nutritional value, though it also affects color and moisture content (Silva et al. 2020). Bagasse contributes positively to the structural integrity and nutritional enhancement of baked goods. Recent research on the use of sugarcane bagasse in donuts has shown that it influences color and texture characteristics. Adding 8.58% bagasse to donuts and frying for 5 min improves the overall quality, with increased redness and decreased lightness and porosity of the crust (Darapoor et al. 2023). The incorporation of bagasse offers the potential for developing fiber‐enriched donut products. Sugarcane bagasse is rich in cellulose, hemicellulose, and ash, making it suitable for various food applications (Table 3).

**TABLE 3:** Physicochemical properties of sugarcane bagasse used for food products.

Parameters	In percentage	References
Cellulose	40%–50%; 26%–47%; 32%–48%; 42%–58.2%	Franck (2005), Jacobsen and Wyman (2002), Jarman (1998), Mahmud and Anannya (2021), Patni et al. (2006), Rankilor (2000), Bilal et al. (2020), and Khatri and Pandit (2022)
Hemicellulose	25%–35%; 19%–23%; 27%–32%; 9.2%–25%	Wyman (1999), Mahmud and Anannya (2021), Franck (2005), Bilal et al. (2020), and Khatri and Pandit (2022)
Ash	1%–5%; 1.5%–5%	Mahmud and Anannya (2021) and Franck (2005)
Lignin	14%–23%; 19–24; 13.4%–20%	Mahmud and Anannya (2021), Franck (2005), Bilal et al. (2020), and Khatri and Pandit (2022)

**TABLE 4:** Comparative summary of maximum acceptable inclusion levels and corresponding nutritional changes.

Food matrix	Typical max acceptable inclusion reported	Typical nutritional changes at/near that level	Sensory/textural summary	References
Cookies/Biscuits	~5% (commonly best) — some studies report acceptability to 10%–15% with processed bagasse or formulation adjustments (Vijerathna et al. 2019)	TDF ↑ markedly (depending on bagasse fiber); fat and energy per 100 g ↓ slightly (dilution); moisture changes depending on water binding (Sangeetha et al. 2011).	5%: minimal sensory loss; ≥ 10%–15%: texture tougher, darker color, possible lower acceptability unless optimized (Vijerathna et al. 2019).	Vijerathna et al. (2019), Sangeetha et al. (2011), Morales‐Tapia et al. (2023), and Vijerathna et al. (2019)
Bread (yeast‐leavened)	**~**7.5%–10% (acceptable if emulsifiers/hydrocolloids are used); without compensating ingredients, sensory/volume drop earlier (Sangnark and Noomhorm 2004).	TDF ↑ (proportional); specific volume ↓; crumb firmer; energy/protein per 100 g ↓ slightly (Sangnark and Noomhorm 2004).	Dough weakening and reduced gas retention; formulation aids can restore acceptable loaf quality (Sangnark and Noomhorm 2004).	Sangnark and Noomhorm (2004), Gil‐López review (data), and Sangnark and Noomhorm (2004)
Cake (oat‐banana)	3%–6% (3–6 g/100 g solids tested)	↑ TDF: possible changes in crumb structure and loaf volume; improved shelf stability with emulsifiers.		Silva et al. (2020)
Noodles/Pasta	5% often best for acceptability; 10%–15% possible — 15% gives clear “high‐fiber” labelling (Lau et al. 2022).	TDF ↑ strongly (e.g., control 3.4% → 13.9% at 15% SB); dialysable glucose ↓ (potential glycaemic benefit) (Lau et al. 2022).	Higher firmness, darker color; acceptability declines with % bagasse, but can be acceptable at 10%–15% in certain markets (Lau et al. 2022).	Lau et al. (2022) and Gil‐López et al. (2019a, 2019b)
Cakes/Quick breads	~3%–6% (reported as 3–6 g/100 g solids in some studies) for maintaining softness and consumer acceptance (Silva et al. 2020).	TDF ↑; crumb tends to become denser at higher inclusion, energy ↓ modestly (Ojha and Verma 2023).	3%–6%%: minimal change; > 6%–10%: denser, poorer crumb and lower acceptability (Silva et al. 2020).	Silva et al. (2020); other small‐scale cake studies.
Other (specialty bars, extrudates)	Variable; often 5%–15% depending on matrix and process (Morales‐Tapia et al. 2023).	TDF ↑; functional claims (high‐fiber) possible at higher inclusions (Gil‐López et al. 2019b).	Sensory depends on matrix; extruded snacks may better tolerate high fiber with a suitable formulation (Gil‐López et al. 2019a, 2019b).	Selected pilot works and product trials (Morales‐Tapia et al. 2023).

**TABLE 5:** Sugar cane bagasse impact on different livestock.

Number	Livestock's	Application/benefits	References
1	Goats	Research comparing sugarcane bagasse to elephant grass hay in goats showed that while bagasse improved the digestibility of dry matter and crude protein, elephant grass hay led to better carcass characteristics. Intact goats also showed superior performance compared to castrated ones.	Campelo‐Lima et al. (2022)
2	Buffaloes	In lactating buffaloes, replacing barley straw with up to 40% sugarcane bagasse improved nutrient digestibility and milk production. This indicates that bagasse can be an effective roughage source, enhancing rumen fermentation and overall milk yield.	Abd El‐Mola and Elnesr (2023)
3	Dairy cows	Sugarcane bagasse can replace wheat straw in dairy cow diets without affecting milk production or chewing behavior. However, its inclusion at higher levels (45%–50%) can replace diets based on spineless cactus, maintaining milk production at 12 kg/d but decreasing overall nutrient intake and digestibility at higher levels.	de Almeida et al. (2018) and Molavian et al. (2020)

**TABLE 6:** Physicochemical properties used for fish feed.

Parameters	In a dry base	References
Protein	1%–4% and 1%–5%	Suryaningrum and Samsudin (2021) and Suryaningrum (2022)
Lipids	< 4%	Suryaningrum (2022) and Suryaningrum and Samsudin (2021)
Ash	2%–8% and 2%–10%	Suryaningrum and Samsudin (2021) and Suryaningrum (2022)
Crude fiber	20%–38% and 20%–40%	Suryaningrum (2022) and Suryaningrum and Samsudin (2021)
Nitrogen‐Free Extract (NFE)	52%–61% and 50%–60%	Suryaningrum and Samsudin (2021) and Suryaningrum (2022)
Lignin	11%–27%	Suryaningrum and Samsudin (2021)
Cellulose	26%–49%	Suryaningrum and Samsudin (2021)
Hemicellulose	16%–33%	Suryaningrum and Samsudin (2021)

### 3.5 Bioactive and Nutritional Potential of Sugarcane Bagasse

#### 3.5.1 Nutritional Composition and Health‐Promoting Properties

Sugarcane bagasse (SCB), the fibrous residue remaining after juice extraction from sugarcane, is increasingly recognized as a valuable by‐product with substantial nutritional and functional potential. Chemically, it is rich in dietary fiber, antioxidants, and essential minerals, which contribute to numerous health benefits. The high fiber content enhances digestive health by improving gut motility, preventing constipation, and lowering serum cholesterol levels. Moreover, the presence of antioxidants in SCB supports immune function and helps prevent infections by reducing oxidative stress and inflammation (Hassan et al. 2024). Additionally, antioxidants in SCB strengthen immune function by mitigating oxidative stress and inflammation (Juliantoni et al. 2023). From a nutritional standpoint, SCB is considered a low‐calorie ingredient that supports weight management and metabolic health (Juliantoni et al. 2023).

Chemically, SCB contains approximately 40%–50% cellulose, 30%–35% hemicellulose, and 10%–14% lignin (Dimopoulou and Kontogiorgos 2020). Soluble dietary fibers extracted under alkaline conditions display promising physiological functions, improving gut health and lowering glycemic response (Chong et al. 2019). Incorporating SCB into food systems, such as oat–banana cakes and noodles, enhances total dietary fiber content without compromising sensory properties (Lau et al. 2022; Silva et al. 2020).

Despite its advantages, SCB's high lignin content can limit digestibility and functionality, requiring pretreatments (e.g., alkaline, enzymatic, or steam) to improve its food applicability (Wahlang et al. 2012). Nonetheless, moderate substitution levels (~10%) have shown acceptable results in baked products (Sangeetha et al. 2011).

#### 3.5.2 Bioactive Compounds and Antioxidant Activity of Bioactive Fractions

Sugarcane bagasse contains phenolic acids, flavonoids, and lignin, which exhibit antioxidant, antimicrobial, and anti‐inflammatory effects (Miao et al. 2016). Girardi et al. (2019) reported exceptionally high polyphenol content (728,201 ± 58.21 mg GAE/100 g) and flavonol levels (325,143 ± 19.03 mg RE/100 g), coupled with strong antioxidant capacity. Enzymatic‐assisted extraction increases phenolic yield by ~50%, identifying p‐coumaric and ferulic acids as dominant compounds (Coniglio et al. 2022).

##### 3.5.2.1 Pharmaceutical and Medicinal Applications

Historically, sugarcane and its by‐products, including bagasse, have held an important place in traditional medicine for treating ailments such as wounds, ulcers, inflammation, pain, fever, cough, and diabetes. The medicinal potential of SCB is primarily attributed to its bioactive compounds, notably phenolic acids, flavonoids, and lignin, which exhibit a wide spectrum of biological activities including anti‐inflammatory, antibacterial, antifungal, antiviral, and antidiabetic effects (Miao et al. 2016).

Recent advancements have further explored SCB's pharmaceutical potential, particularly in the field of nanocellulose‐based biomaterials. Nanocellulose extracted from sugarcane bagasse has been found to possess intrinsic antibacterial properties, making it a promising candidate for medical and drug delivery applications. In one study, nanocellulose extracted from SCB achieved a 55.6% yield and demonstrated a 53.12% bacterial inhibitory rate, validating its potential for use in wound dressings and biomedical devices (Charoensopa et al. 2024).

Furthermore, SCB has shown promise as an adjuvant therapy for improving the quality of life in stable chronic obstructive pulmonary disease (COPD) patients, possibly due to its antioxidant and anti‐inflammatory mechanisms (Miao et al. 2016). These findings highlight the emerging therapeutic relevance of SCB and the need for continued pharmacological validation and safety assessments.

##### 3.5.2.2 Nanocrystalline Cellulose (NCC)

Nanocrystalline cellulose derived from SCB exhibits high crystallinity, biocompatibility, and thermal stability. It serves as a biodegradable pharmaceutical excipient with superior disintegration and dissolution properties in oral formulations (Dabhi et al. 2025; Mishra 2024). Moreover, SCB‐derived NCC demonstrates intrinsic antibacterial activity, achieving a 53.12% bacterial inhibition rate, suggesting applications in wound dressings and biomedical coatings (Charoensopa et al. 2024).

##### 3.5.2.3 Lipid Extracts and Metabolic Health

Sugarcane bagasse also yields lipid fractions rich in biologically active compounds, including octacosanol, phytosterols, and triterpenoids (Pereira et al. 2024; Teixeira et al. 2021). These compounds are known for their anti‐hypercholesterolemic, anti‐hyperglycemic, antioxidant, and anti‐inflammatory effects, which collectively contribute to mitigating metabolic syndrome‐associated disorders (Pereira et al. 2024; Teixeira et al. 2021). The extraction of these lipids commonly employs environmentally friendly techniques such as ethanol extraction, molecular distillation, and supercritical CO_2_ extraction to ensure both efficiency and sustainability (Teixeira et al. 2021). Their inclusion in nutraceutical formulations holds promise for the development of functional supplements aimed at improving lipid and glucose metabolism.

##### 3.5.2.4 Antimicrobial and Multifunctional Potential

In addition to antioxidant and metabolic benefits, SCB exhibits considerable antimicrobial potential. Studies demonstrate that both its cellulose and lignin components can be engineered into antimicrobial materials. For instance, eugenol‐encapsulated cellulose composites derived from SCB inhibited *Staphylococcus aureus* with inhibition zones of up to 20.47 mm, highlighting its application in antimicrobial coatings and packaging (Hernández‐López et al. 2024). Similarly, phenolic compounds in sugarcane molasses, a related by‐product, exhibited antibacterial activity against *E. coli* and *Salmonella*, producing inhibition zones between 8.82 and 25.05 mm (Shafiqa‐Atikah et al. 2020). The presence of garlic and tannic acids was identified as the main contributor to these effects. Furthermore, lignin isolated from SCB effectively inhibited *Staphylococcus epidermidis* growth and presented a favorable cost‐performance ratio relative to commercial antimicrobial agents (Sunthornvarabhas et al. 2019). Such multifunctional activities spanning antioxidant, anti‐inflammatory, and antimicrobial mechanisms demonstrate the versatility of SCB‐derived extracts for pharmaceutical and biomedical applications.

### 3.6 Applications of Bagasse for Animal Feed

Sugarcane bagasse, a by‐product from sugar factories, is a high‐fiber material often used in animal feed (Table 5). Research has demonstrated that treating bagasse with cow feces and Effective Microorganism‐4 (EM‐4) enhances its crude protein content while reducing crude fiber. This makes it a viable feed option, especially when mixed with other ingredients to ensure balanced nutrition for livestock. By recycling bagasse, both the sugar and aquaculture industries can benefit from reduced waste and additional revenue (Juliantoni et al. 2023; Suryaningrum 2022). Fermentation with *Pleurotus florida* has improved bagasse's dry matter, fiber content, protein levels, and digestibility, making it a promising alternative roughage source for ruminants (Mahmood Molaei Kermani et al. 2019). Similarly, enzymatic treatments using *Bacillus subtilis* have enhanced bagasse's protein content and reduced lignin, making it more suitable for fish feed (Table 6) (Suryaningrum and Samsudin 2021). Processing bagasse with oyster mushrooms has significantly improved its nutritional profile, making it a more digestible and palatable feed option for ruminants. In in vivo studies, this treatment has led to better feed intake, digestibility, and overall performance in animals (Mahmood Molaei Kermani et al. 2019).

Utilizing sugarcane bagasse in animal feed offers a dual benefit by managing agricultural waste from sugar production while providing a cost‐effective and sustainable feed ingredient. This practice aligns with circular economy principles in agriculture by reducing waste and contributing to environmental sustainability (Juliantoni et al. 2023). Research has demonstrated the versatility of sugarcane bagasse as a feed component across various livestock species, improving feed quality, digestibility, and overall animal performance. For example, steam‐exploded sugarcane bagasse, after appropriate pretreatment, has shown potential as a high‐energy feedstock for beef cattle by enhancing digestibility and energy availability (Chen et al. 2019). Furthermore, its inclusion in livestock diets has been associated with improved nutrient digestibility and increased milk production, offering a viable alternative to traditional roughage sources such as wheat straw and barley straw. However, it is important to note that while bagasse improves digestibility, higher inclusion levels may lead to reduced overall nutrient intake and digestibility. In comparative studies, elephant grass hay was found to produce better carcass traits in goats than sugarcane bagasse (Table 5) (Abd El‐Mola and Elnesr 2023; Campelo‐Lima et al. 2022; de Almeida et al. 2018; Molavian et al. 2020).

In addition to its applications in animal nutrition, sugarcane bagasse is increasingly used in biodegradable packaging materials, particularly for food contact applications. Ensuring compliance with regulatory standards such as EU Regulation No. 10/2011 and FDA Title 21 is essential for the safe use of bagasse‐derived materials in packaging, confirming they meet safety requirements for direct food contact (Stroescu et al. 2024). Moreover, certification under ASTM D6400 and EN 13432 validates the biodegradability and compostability of sugarcane bagasse products, facilitating broader market acceptance and regulatory approval. To holistically assess the environmental footprint of these applications, Life Cycle Assessment (LCA) methodologies, including those outlined in the ISO 14040 series, play a critical role. These tools provide standardized approaches to evaluating the sustainability and environmental impacts of sugarcane bagasse throughout its lifecycle, supporting informed decision‐making in product development and policy formulation (Stroescu et al. 2024). Overall, sugarcane bagasse stands out as a multifunctional and eco‐friendly material with proven benefits in both animal feed and biodegradable packaging sectors.

Extracted lignin from sugarcane bagasse using an alkaline method, yielding 21.3% lignin characterized by 63.8% carbon and 0.6% ash content (Fangueiro et al. 2023). This lignin demonstrated prebiotic potential by promoting beneficial bacteria such as *Lactobacillus* spp. and *Bifidobacterium* spp., making it a suitable additive for poultry feed. At 0.5% and 1.0% concentrations in feed, it improved broiler chickens' body weight gain, feed conversion ratio, and European efficiency factor. European Efficiency Factor (EEF) is a widely used performance index in poultry production to evaluate the overall efficiency of broiler chicken growth, considering livability, body weight, feed conversion ratio (FCR), and age at slaughter. The authors claimed that lignin supplementation also enhanced gut health, microbiota composition, immune response, and antioxidant capacity, indicating its potential as a valuable prebiotic additive in poultry feed (Fangueiro et al. 2023). Extracted lignin from bagasse has shown prebiotic potential, promoting beneficial gut bacteria and improving poultry performance. The inclusion of bagasse lignin in poultry feed enhances body weight gain, feed conversion, and overall health (Fangueiro et al. 2023). Ammonization and fermentation with *Trichoderma harzianum* can enhance bagasse quality for animal feed. Studies showed that 10% sago flour addition during fermentation improved organic matter digestibility and dry matter digestibility. Length of incubation did not significantly affect crude fiber content but did affect crude protein content. The authors recommend using 10% sago flour for optimal fermentation results (Samadi et al. 2016).

Research has enhanced the suitability of bagasse as a component of fish feed, making it viable for animal consumption. Including bagasse in feed formulations can help reduce costs, particularly in aquaculture, where affordable feed options are essential (Suryaningrum 2022; Suryaningrum and Samsudin 2021). In aquaculture, processed bagasse can be incorporated into fish feed to lower costs and support sustainable operations.

### 3.7 Other Applications of Sugarcane Bagasse

Sugarcane bagasse can be utilized for diverse applications like adsorbent, ion exchange resin, briquette, ceramic, concrete, cement, and polymer composite, as highlighted in the research paper (Mahmud and Anannya 2021). Studies demonstrate its effectiveness in removing pollutants from wastewater. For instance, bagasse can efficiently adsorb lead ions from aqueous solutions, showing a maximum adsorption capacity of 60.24 mg/g for Pb (II) when activated through CO_2_ physical activation (Somyanonthanakun et al. 2023). This capability is crucial for wastewater purification processes, providing a cost‐effective alternative to traditional adsorbents. Similarly, it has been successfully used to remove methylene blue dye up to 86% under optimal conditions, such as a pH of 6 and an equilibrium contact time of 30 min (Bwankwot et al. 2024).

Additionally, bio‐activated carbon derived from bagasse achieved a high removal efficiency of 99.9% for chromium (III) under specific conditions (Apriani et al. 2023). The potential of sugarcane bagasse as an effective and cost‐efficient material for water treatment exists. In the construction industry, sugarcane bagasse ash (SCBA) and bagasse‐derived materials are being explored as sustainable alternatives to traditional materials (Figure 7). Sugarcane bagasse ash, with its high silica content, has been utilized as a partial replacement for cement in concrete production. Research indicates that up to 20% SCBA replacement enhances concrete strength (Aravind and Raj 2022). Furthermore, combining sugarcane bagasse ash with other materials such as ceramic tile waste (CTW) optimizes concrete properties, with a 10% SCBA and 10% CTW replacement showing improved compressive strength (Jumani et al. 2023). These applications not only address the waste disposal issue but also improve the performance of concrete. Incorporating SCBA as a partial cement substitute has been shown to achieve a maximum compressive strength of 29.31 MPa when combined with ceramic tile waste (CTW) (Jumani et al. 2023).

**FIGURE 8:**
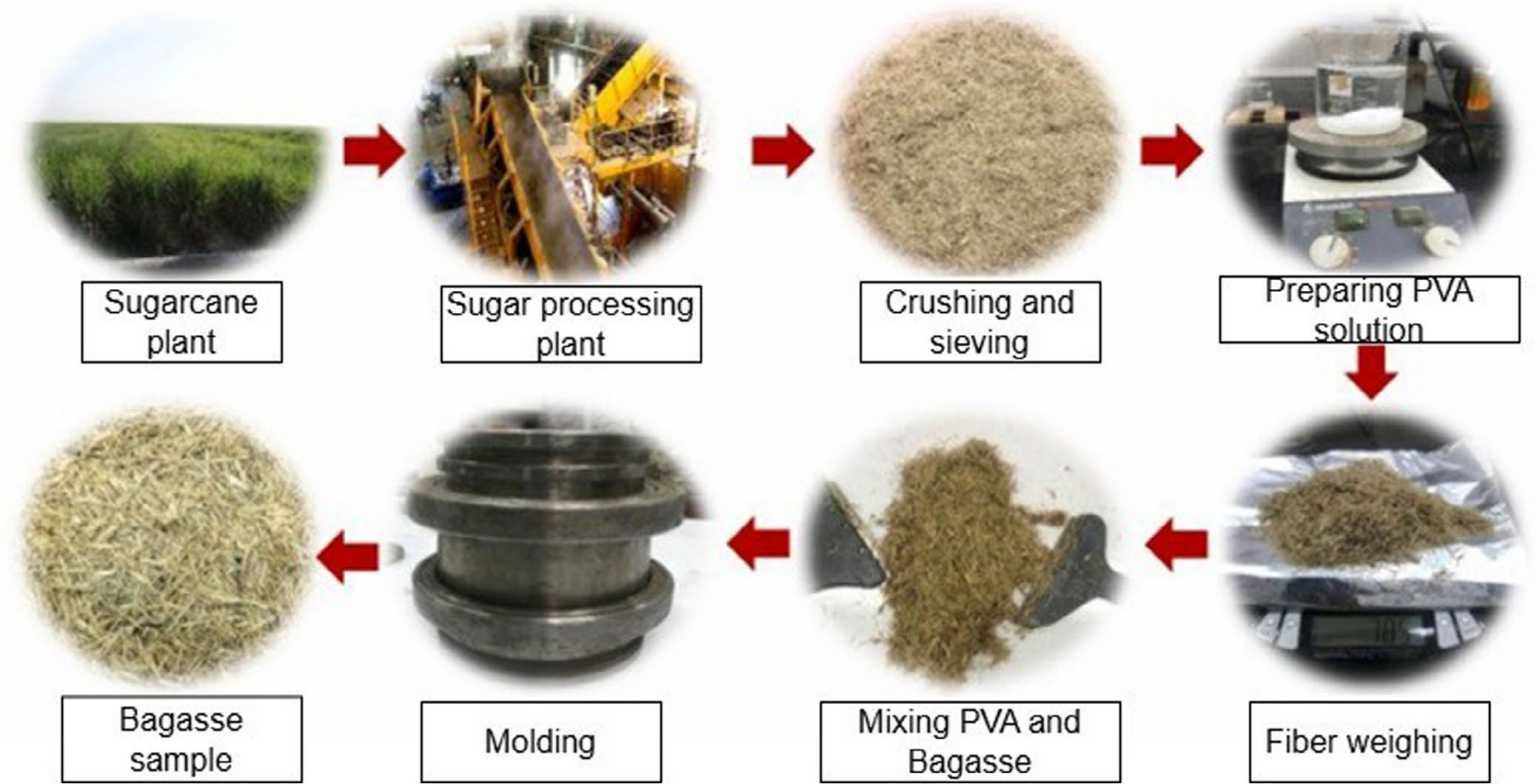
Different preparation stages of sugarcane bagasse waste samples for the development of thermal insulation and sound‐absorbing building materials.

Sugarcane bagasse ash is increasingly being considered as a filler in polymer matrices. The ash's silica content makes it a promising additive in rubber composites, potentially reducing reliance on petroleum‐based fillers. Studies using techniques such as X‐ray diffraction (XRD), Fourier‐transform infrared spectroscopy (FTIR), and scanning electron microscopy with energy‐dispersive X‐ray spectroscopy (SEM–EDX) confirm the ash's suitability as a sustainable and cost‐effective filler material (Seroka et al. 2022). This innovative application supports more environmentally friendly industrial practices and enhances the performance of composite materials. Sugarcane bagasse fibers are increasingly used in polymer composites, emerging as alternatives to new technological materials, such as thermal insulation and sound‐absorbing building materials (Figure 8) (de Paiva et al. 2019; Guna et al. 2019; Marichelvam et al. 2021; Ramlee et al. 2021; Mehrzad et al. 2022). Studies have investigated the reinforcement of various polymer matrices, both natural and synthetic, with bagasse fibers. Specifically, fiber loadings of 30%, 35%, and 40% wt.% of untreated and treated fibers (5% NaOH, 30 min, 30°C, liquor ratio of 20:1) in an epoxy resin matrix have been analyzed (Mehrzad et al. 2022) (Figure 8).

Studies also suggest that sugarcane bagasse‐derived biochar can be used to create engineered cementitious composites with enhanced strength and ductility (Amin et al. 2020). Another advantage of sugarcane bagasse is its ability to be efficiently converted into activated charcoal through carbonization and activation processes. This activated charcoal shows great potential for adsorbing chemical oxygen demand (COD) from Sasirangan wastewater. According to a study by Noor et al. (2022), the optimal conditions for maximum COD reduction (up to 95.37%) in sugarcane bagasse were found to be a pH of 5, a contact time of 90 min, and a charcoal dose of 5 g/L. Studies indicated that the dose of bagasse‐activated charcoal plays a crucial role in COD removal efficiency, with 5 g/L identified as the maximum effective dose (Noor et al. 2022).

## 4 Environmental and Economic Impacts of Sugarcane Bagasse

### 4.1 Environmental Benefits

Sugarcane bagasse (SCB), a fibrous byproduct of the sugar industry, offers multiple environmental advantages when valorized rather than discarded. One of its most significant applications lies in environmental remediation. Compared to synthetic adsorbents, bagasse‐based materials provide an economical and eco‐friendly alternative for removing pollutants from wastewater. Studies have demonstrated that untreated and modified bagasse effectively adsorb dyes such as methylene blue and heavy metals like Pb (II), Ni (II), and Cr (III), achieving up to 99.9% removal efficiency under optimal conditions (Apriani et al. 2023; Bwankwot et al. 2024; Ighalo et al. 2023). The cellulose and lignin components in bagasse enhance its adsorption capacity, while physical, chemical, and biological modifications further improve performance. For instance, biochar derived from bagasse has shown phenol removal efficiencies as high as 96.1% (Hamzah et al. 2022; Saini et al. 2022).

In addition to wastewater treatment, the transformation of sugarcane bagasse ash into filler material for rubber, polymer, and construction composites contributes to sustainable waste utilization. This conversion reduces landfill burden and reliance on non‐renewable raw materials, thereby lowering the environmental footprint of industrial production (Mizar et al. 2020; Seroka et al. 2022). Furthermore, the conversion of bagasse into bioethanol and bioenergy provides a renewable and clean energy alternative to fossil fuels. This not only mitigates greenhouse gas emissions but also aligns with global decarbonization goals (Awasthi et al. 2024; Mizar et al. 2020). Bioethanol derived from SCB exhibits higher combustion efficiency and cleaner burning characteristics than gasoline, reducing particulate matter emissions and environmental pollution (Esquerre Verástegui 2016; Mizar et al. 2020).

Beyond energy production, bagasse pulp has also been applied in manufacturing biodegradable products such as cups, plates, and packaging materials. These materials, being biodegradable and free from toxic additives, serve as sustainable alternatives to petroleum‐based plastics, thereby reducing plastic pollution and associated health risks (Hossam and Fahim 2023).

In agriculture and aquaculture, sugarcane bagasse demonstrates functional environmental benefits. In aquaculture systems, dietary inclusion of SCB enhances growth performance, immunity, and antioxidant gene expression in shrimp larvae, improving zootechnical indices and disease resistance (Hassan et al. 2024). In crop production, SCB acts as a natural biosorbent, reducing heavy metal concentrations in contaminated soils and water sources (Ndebele 2023). For instance, its application has been shown to alleviate arsenic toxicity in wheat by improving physiological responses and reducing oxidative stress (El‐Shehawi et al. 2022). Similarly, when incorporated as roughage in livestock feed, SCB improves nutrient digestibility and growth performance in beef calves, contributing to more sustainable animal production systems (Singer and Marwan 2019).

Overall, these findings highlight that the environmental significance of sugarcane bagasse extends beyond energy generation. Its applications in pollution control, biodegradable material production, and sustainable agriculture collectively contribute to waste minimization, emission reduction, and resource circularity, key elements of environmental sustainability.

### 4.2 Economic Benefits

**FIGURE 9:**
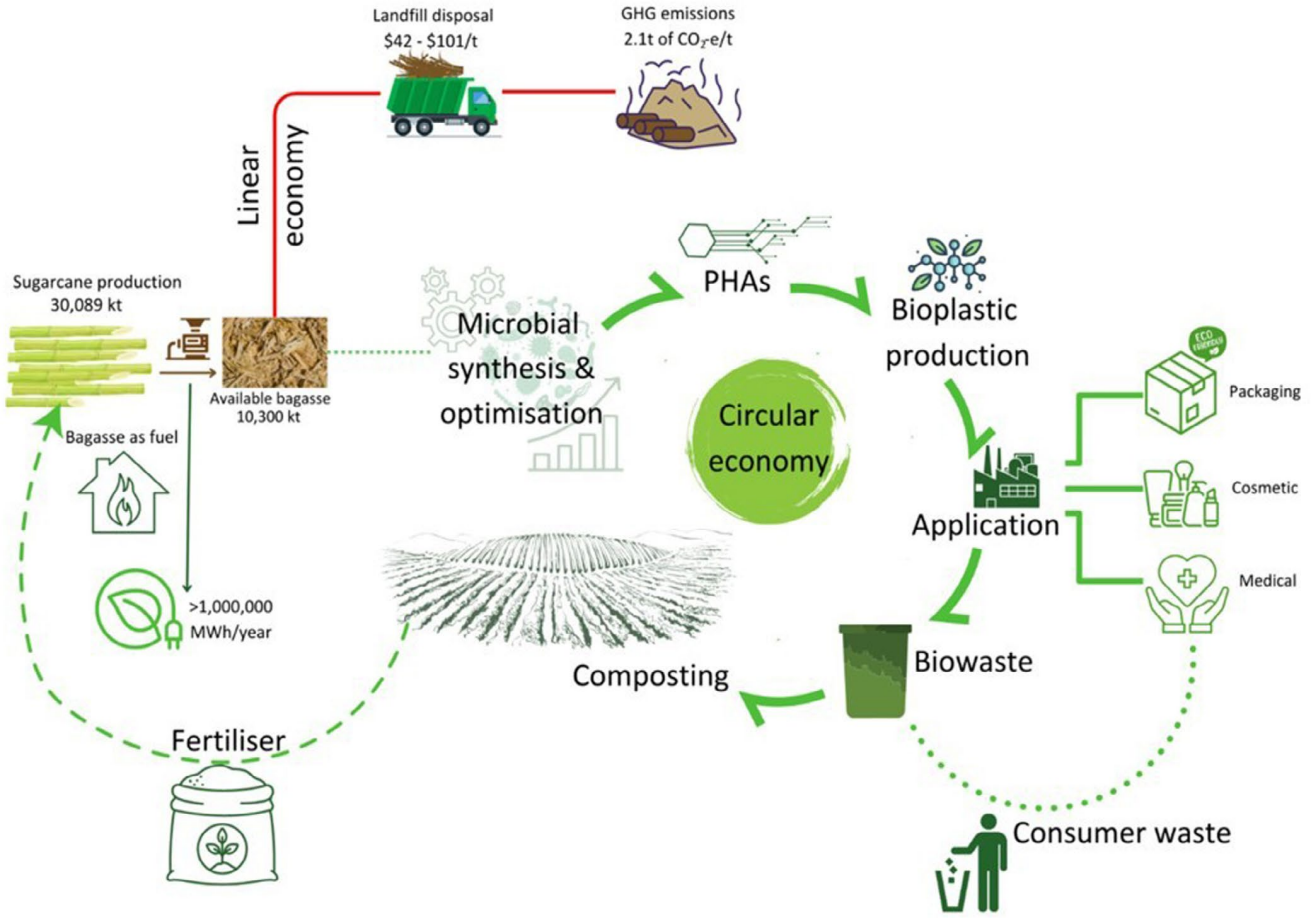
A comparison between linear and circular economy models related to bagasse production in Australia. The available bagasse indicates the portion of bagasse that remains after fulfilling energy needs.

Sugarcane bagasse provides remarkable economic benefits through its versatile industrial applications, contributing significantly to sustainable development and national economic growth. It serves as a valuable by‐product that supports renewable energy production, material innovation, and agricultural productivity, thereby transforming what was once considered waste into an economically valuable resource (Awasthi et al. 2024; Mizar et al. 2020; Triyani et al. 2023). One of the most notable economic contributions of bagasse lies in its use as a renewable energy source. For example, in Australia, sugarcane bagasse functions as a carbon‐neutral biofuel that generates over one million MWh of electricity annually in a cost‐effective manner, where about 56% of the produced energy powers sugar mills and the remaining 44% is supplied to the national grid, sufficient to provide electricity to approximately 135,000 households (Figure 9) (Sugar Mills a Source 2012). This not only reduces dependence on fossil fuels but also promotes energy self‐sufficiency and rural economic stability.

Moreover, sugarcane bagasse ash (SCBA) holds considerable industrial value due to its high amorphous silica content, making it an excellent filler material in rubber, polymer, and composite manufacturing. Such applications reduce reliance on petroleum‐based materials, lower production costs, and foster environmentally sustainable manufacturing practices (Seroka et al. 2023). Similarly, incorporating SCBA into concrete enhances the mechanical and thermal properties of construction materials while simultaneously reducing CO_2_ emissions and overall energy consumption, offering both environmental and economic advantages (Philip et al. 2021; Sharma and Gurdaspur 2019; Tripathy and Acharya 2022).

In addition, the conversion of sugarcane bagasse into bioethanol provides a cost‐effective and renewable alternative to fossil fuels. This process decreases dependence on imported petroleum, improves national energy security, and generates employment opportunities throughout the cultivation, processing, and distribution chain (Triyani et al. 2023). Compared to gasoline, bioethanol from bagasse also has a lower carbon footprint, aligning economic growth with environmental sustainability. Furthermore, the production of biodegradable packaging and tableware from bagasse has emerged as a commercially viable alternative to petroleum‐based plastics. These eco‐friendly products not only meet the increasing market demand for sustainable materials but also offer producers greater economic stability by avoiding the price volatility and regulatory costs associated with conventional plastics (Hossam and Fahim 2023).

Agriculturally, sugarcane bagasse also plays a crucial economic role when used as an organic soil amendment or livestock feed substitute. It helps reduce input costs while maintaining productivity and quality. Bagasse‐based feed is both affordable and nutritious, improving animal health, whereas its application as an organic amendment enhances soil fertility and reduces dependence on costly synthetic fertilizers (Singer and Marwan 2019). Despite these benefits, improper disposal or inefficient burning of bagasse can lead to environmental issues such as air pollution and greenhouse gas emissions. However, adopting sustainable management techniques such as controlled combustion and efficient recovery systems can mitigate these drawbacks while maximizing their economic potential (Kautzar et al. 2020; Okibe et al. 2023; Paola et al. 2018; Rainey et al. 2012).

Overall, the economic valorization of sugarcane bagasse strengthens industrial efficiency, promotes rural employment, and advances the circular bioeconomy by converting agricultural residues into high‐value products. Through its integration in renewable energy generation, industrial manufacturing, and agriculture, sugarcane bagasse demonstrates its vital dual role as both an economic driver and an environmental safeguard, fostering a more sustainable and resilient global economy (Awasthi et al. 2024; Seroka et al. 2022).

## 5 Applications of Sugar Bagasse on Plant Growth and Soil Fertility

The use of sugarcane bagasse in agriculture has shown substantial benefits for plant growth and soil fertility. Research indicates that incorporating sugarcane ashes and biosolids into the soil enhances bean crop production by supplying essential nutrients and improving soil organic matter content (Ferreira et al. 2023). Additionally, applying ash doses boosts the initial growth of pigeon peas, increases chlorophyll content, and enhances soil fertility parameters such as pH, phosphorus (P), potassium (K), and base saturation (Rubio et al. 2023). Innovative applications include the development of a nanocomposite fertilizer synergist from sugarcane bagasse, which reduces nutrient loss, enhances sugarcane yield, and improves soil physicochemical properties, offering a sustainable approach to cultivation (Zhao et al. 2023). Moreover, a slow‐release iron (Fe) fertilizer hydrogel derived from sugarcane bagasse has also proven effective in gradually supplying nutrients to crops, improving nutrient use efficiency, and reducing environmental pollution (Bharaani Sri et al. 2023).

Sugarcane bagasse effectively enhances plant growth through various mechanisms. It serves as a valuable substrate amendment, improving crop productivity by managing substrate pH and fertility dynamics (Thiessen et al. 2023). Studies have shown that sugarcane bagasse can mitigate arsenic toxicity in plants, leading to increased shoot biomass, enhanced chlorophyll synthesis, and improved membrane stability, while reducing oxidative stress and lipid peroxidation (El‐Shehawi et al. 2022). Furthermore, sugarcane bagasse is an economical medium for supporting fungal growth in laboratory settings, showcasing its nutrient‐rich composition and capacity to stimulate rapid fungal development (Abdullahi et al. 2023). Field trials have demonstrated that using sugarcane bagasse as a soil amendment can significantly increase sugarcane biomass and sugar yield, attributed to enhanced silicon supply and plant nutrition (Xu et al. 2021).

Sugarcane bagasse enhances plant growth by activating the soil, improving water infiltration, promoting nutrient development, and removing heavy metals. It stimulates microbial growth and can be used as a valuable soil amendment (Olusoji David 2022). As a nutrient‐rich resource, sugarcane bagasse contains nitrogen (N), phosphorus (P), potassium (K), and other components like lignin, cellulose, hemicellulose, and ash. This composition is crucial for enriching soil fertility and supporting plant development. Significant nutrients are exported with the harvested stalks in sugarcane production; replenishing these nutrients through organic sources like bagasse is essential (Cantarella and Rossetto 2014). Utilizing sugarcane bagasse in growth media can enhance plant nutrient uptake, particularly phosphorus, potassium, calcium, and magnesium, leading to improved growth and yield in crops like lettuce (Jayasinghe 2012). Sugarcane bagasse enhances plant growth by increasing soil organic matter, water‐holding capacity, nutrient accumulation, and first‐year sugarcane biomass and sugar yield, with the 10 cm application rate showing the most significant benefits. Bagasse application increases soil organic matter, water‐holding capacity, and nitrogen accumulation, and a higher application rate is recommended for increased sugarcane biomass yield (Bhadha et al. 2023). Sugarcane bagasse ash application improves soil fertility by increasing micronutrients and porosity, reducing bulk density, and enhancing *Capsicum frutescens* yield, particularly with a 40%–60% application level (Bhushan 2023). Adding sugarcane bagasse ash to greenhouse media enhances plant growth by providing nutrients and reducing production costs, with optimal benefits observed at a 25% inclusion level for bean and Chinese kale seedlings. However, levels above 50% are not recommended due to adverse effects (Bhushan 2023). Sugarcane bagasse ash also improves squash and cantaloupe seedling growth by enhancing stalk lengths, fresh weights, and dry weights, with optimal results observed at 75% and 25% ash levels, respectively (Webber III et al. 2015). Enriching sugarcane bagasse compost with a bacterial consortium optimizes areca nut production by improving soil fertility and enhancing microbial community structure, thereby promoting plant growth effectively (Liu et al. 2023).

## 6 Challenges and Limitations in the Production of Sugarcane Bagasse Products

The valorisation of sugarcane bagasse (SCB) into high‐value products such as biofuels, biochemicals, fiber‐rich foods, and biomaterials presents substantial opportunities for sustainable industrial development. However, large‐scale implementation is constrained by a combination of technical, economic, compositional, and environmental challenges.

One of the key challenges in producing sugarcane bagasse products is the variable nature of the biomass itself. The composition and physicochemical properties of SCB vary depending on cultivar, geographical location, harvest season, and processing conditions (Wang et al. 2022). This variability complicates process optimization and makes it difficult to maintain consistent product quality. For instance, fluctuations in cellulose, hemicellulose, lignin, ash, and moisture content can significantly affect enzymatic efficiency, pretreatment chemistry, and final product characteristics. Templeton et al. (2016) and Andrade et al. (2017) reported that compositional variations in raw bagasse directly influence sugar recovery and hydrolysis efficiency. Differences in particle size, fiber distribution, and extractive content further impact reactor performance and quality control, making feedstock standardization a critical industrial barrier.

Another significant limitation lies in the technological and economic barriers associated with the efficient conversion of sugarcane bagasse into value‐added products (Meghana and Shastri 2020). Although various biochemical and thermochemical conversion technologies have been developed, their commercialization remains restricted by high capital costs, technical complexity, and the need for process optimization (Eggleston and Lima 2015; Meghana and Shastri 2020). The production chain involves several energy‐ and resource‐intensive unit operations, including pretreatment, enzymatic hydrolysis, and downstream purification.

The pretreatment stage, involving delignification and fractionation, is essential for disrupting the lignocellulosic matrix of cellulose, hemicellulose, and lignin. However, it requires substantial energy, water, and chemical inputs and often leads to the formation of inhibitory by‐products that are costly to remove (Balaguer Moya et al. 2022). Subsequent enzymatic hydrolysis (saccharification) converts polysaccharides into fermentable sugars but incurs major costs due to the high price of cellulolytic enzymes, long reaction times, and enzyme inhibition effects (Visser et al. 2015). Even with advanced enzyme systems such as lytic polysaccharide monooxygenases (LPMOs), costs and process complexity remain high (Balaguer Moya et al. 2022). Downstream purification and separation—required for lignin removal, sugar recovery, and product concentration—further elevate operational expenses. Although aqueous two‐phase system (ATPS) hydrolysis can enhance separation efficiency, it introduces additional steps that challenge industrial scalability (Bussamra et al. 2020). High solids loading in reactors also causes viscosity and mixing constraints, increasing both capital and operational expenditures.

The recalcitrance of lignocellulosic biomass presents another persistent obstacle. The crystalline cellulose structure and tight lignin carbohydrate complex limit enzyme accessibility and reduce hydrolysis efficiency, even under optimized pretreatment (Bussamra et al. 2020). This recalcitrance, combined with the formation of inhibitory compounds such as phenolics, furans, and organic acids, further impairs enzymatic and microbial performance (Balaguer Moya et al. 2022). In food and nutraceutical applications, these inhibitory compounds, along with potential heavy metals or chemical residues, raise additional safety and regulatory challenges that complicate certification and market entry.

Moreover, several studies have emphasized economic and commercialization barriers in bagasse valorisation. High enzymatic hydrolysis costs, low fermentation yields, and process inefficiencies continue to limit the commercial viability of SCB‐based biofuels (Algayyim et al. 2022; Singh et al. 2021). The cost of cellulose production remains one of the principal bottlenecks (Camassola and Dillon 2014). Despite advances in biocatalyst development and process integration, the commercial deployment of green technologies utilizing bagasse remains limited, requiring further technological improvement for viable industrial applications (Singh et al. 2022).

Beyond biofuels, bagasse has also been explored as a bio‐coagulant precursor for water treatment, but challenges persist related to cost, scalability, and process optimization for practical use (Iwuozor et al. 2023). Similarly, the integration of sustainable biocatalysts such as microbial enzymes for eco‐friendly nutraceutical production in the sugarcane industry presents technical and economic challenges (Korasapati et al. 2023).

From an engineering perspective, infrastructure and supply chain logistics represent additional constraints. Transitioning SCB from its conventional role in energy cogeneration to feedstock for high‐value bioproducts requires major capital investment and the establishment of new biorefinery facilities. Collecting, transporting, drying, and processing bagasse at scale contribute to overall cost and process variability (Singh et al. 2022). The high ash content in bagasse also poses a limitation for fuel pellet production; however, wet torrefaction has been identified as a promising technique to reduce ash levels and enhance economic feasibility (Jarunglumlert et al. 2022).

Finally, the sustainability and environmental implications of SCB utilization must be carefully evaluated. Although sugarcane bagasse is a renewable feedstock, the entire supply chain from cultivation and processing to waste management must be managed holistically to ensure genuine environmental benefits (Eggleston and Lima 2015). Sustainable alternatives to synthetic binders, ensuring adequate mechanical strength, and addressing emissions and disposal issues remain vital considerations (Nyangau et al. 2019).

## 7 Prospects for Bagasse and Biodegradable Alternatives

The future of sugarcane bagasse and other biodegradable alternatives is highly promising, offering opportunities to advance sustainability across energy, agriculture, materials, and food sectors. To fully harness these benefits, future efforts should prioritize technological innovation, economic feasibility, and supportive policy development.

Research should focus on improving the efficiency and environmental performance of bagasse‐based technologies through low‐energy pretreatments, advanced enzymatic hydrolysis, and effective solvent recovery methods. Pilot‐scale trials are needed to validate the mechanical, thermal, and biodegradation properties of nanocellulose and bio‐composites derived from bagasse. In parallel, further investigation into the functional roles of sugarcane bagasse in food systems, particularly its prebiotic, antioxidant, and antimicrobial effects, could unlock new nutritional and health applications.

Moreover, integrating life cycle assessments with techno‐economic analyses will be vital for evaluating scalability and guiding sustainable industrial adoption. Expanding the use of bagasse in biodegradable packaging, animal feed improvement, pollutant removal, and sustainable agriculture can enhance its contribution to a circular bioeconomy. Achieving this vision will require strong collaboration among researchers, industry stakeholders, and policymakers to establish regulatory standards, promote investment, and ensure that sugarcane bagasse evolves from an agricultural residue into a cornerstone of green innovation and sustainable development.

## 8 Conclusion

Sugarcane bagasse, an abundant lignocellulosic byproduct of the sugar industry, holds remarkable potential to advance sustainability through bioenergy generation, green material development, and food system innovation. Its high cellulose and hemicellulose content make it an efficient feedstock for renewable fuels such as bioethanol and biogas, achieving yields of up to 3.7% (v/v) ethanol and 347.6 mL CH_4_/g VS under optimized conditions. These bioenergy pathways contribute significantly to reducing greenhouse gas emissions and reliance on fossil fuels.

Beyond energy applications, bagasse‐derived biodegradable packaging and composites utilize its cellulose‐rich fibers to produce eco‐friendly materials with strong mechanical and barrier properties, providing viable alternatives to petroleum‐based plastics. In the food and feed sectors, the incorporation of bagasse flour can enhance dietary fiber content by more than 30% in baked products, improve gut health, and boost livestock performance, thereby supporting both human and animal nutrition. Despite its promising potential, the large‐scale utilization of sugarcane bagasse continues to be hindered by high production costs, technological limitations, compositional inconsistencies, and insufficient regulatory frameworks governing its use in food and health‐related applications. Moreover, while life‐cycle assessments demonstrate clear environmental benefits, comprehensive techno‐economic analyses and standardized performance benchmarks are still limited.

Future advancement of sugarcane bagasse valorization requires the development of cost‐effective, energy‐efficient, and scalable green processing technologies. Equally important are robust safety assessments, supportive policy frameworks, and cross‐sectoral integration into circular biorefineries. By addressing these challenges, sugarcane bagasse can be transformed from an underutilized residue into a strategic, high‐value feedstock that drives innovation, resource circularity, and climate resilience within a sustainable bioeconomy.

## Author Contributions

Desye Alemu Teferi guided the study and oversaw data collection. Messenbet Geremew Kassa, Mikru Tesfa Belachew, and Eshetie Gelagay Erku, along with the other authors, provided technical comments, revised the manuscript, and approved the final version for publication. Each author's contributions were essential to the success and integrity of the research study.

## Funding

The authors have nothing to report.

## Conflicts of Interest

The authors declare no conflicts of interest.

## Data Availability

The data that support the findings of this study are available from the corresponding author upon reasonable request.
